# Heat stroke–induced liver injury: mechanistic insights, immune dynamics, and emerging therapeutic targets

**DOI:** 10.3389/fimmu.2026.1864759

**Published:** 2026-10-30

**Authors:** Ning Li, Sujing Liu, Xinyu Hu, Xiaoqing Wu, Mengna Song, Fang Luan, Youyong Tang, Yong Wang

**Affiliations:** 1 Department of Clinical Laboratory, Shandong Provincial Hospital Affiliated to Shandong First Medical University, Jinan, Shandong, China; 2 Department of Oncology, Shandong Provincial Third Hospital, Shandong University, Jinan, Shandong, China

**Keywords:** gut–liver axis, heat stroke, liver injury, oxidative stress, systemic inflammation

## Abstract

Heat stroke is a life-threatening heat-related emergency that often leads to multiple organ dysfunction syndrome (MODS), with liver injury among its most prominent manifestations. HS-associated acute liver injury (HS-ALI) is a major determinant of prognosis, yet its pathogenesis remains incompletely understood. Current evidence suggests that HS-ALI results from the interplay of direct thermal stress, systemic inflammation, oxidative imbalance, coagulation dysfunction, and regulated cell death. Emerging experimental studies have implicated pyroptosis, ferroptosis, necroptosis, and gut–liver immune crosstalk in HS-associated tissue injury; however, the strength of evidence varies considerably among these mechanisms, and direct validation in human HS-ALI remains limited. Accordingly, these pathways should be considered potential contributors to disease progression rather than uniformly established mechanisms.

## Highlights

- Heat stroke–associated liver injury arises from coupled thermal, vascular, and immune insults.
- Gut barrier breakdown and immunothrombosis amplify hepatic endothelial dysfunction.
- Oxidative stress and mitochondrial failure converge on hepatocellular metabolic collapse.
- Ferroptosis, pyroptosis, necroptosis, and autophagy-related responses form an interconnected network of cellular stress responses and regulated cell death.

## 1 Introduction

The growing frequency of extreme heat events associated with global climate change has become an important public health challenge worldwide (1). Among heat-related illnesses, heat stroke (HS) represents the most severe clinical manifestation, characterized by a rapid elevation of core body temperature (typically ≥40 °C), central nervous system dysfunction, and systemic inflammatory responses, often accompanied by progressive multiple organ dysfunction (MODS) and high mortality. Clinically, HS can be broadly classified into classic heat stroke (CHS), which mainly occurs during passive exposure to environmental heat and disproportionately affects elderly individuals and those with impaired thermoregulatory capacity, and exertional heat stroke (EHS), which typically develops in physically active individuals during strenuous exercise (2). Although both conditions share common pathological features, differences in initiating stressors, host susceptibility, inflammatory responses, and organ injury patterns suggest that HS represents a heterogeneous clinical syndrome rather than a uniform disease entity.

Among the organs affected by HS, the liver is considered one of the most vulnerable organs and a major determinant of clinical outcome (3). Severe HS-associated liver injury (HS-ALI) may rapidly progress from acute hepatocellular injury to acute liver failure (ALF), occasionally requiring emergency liver transplantation. Data from the Acute Liver Failure Study Group indicate that HS-related ALI/ALF was associated with marked elevation of aminotransferases, coagulopathy, and substantial mortality, highlighting the clinical significance of hepatic involvement during HS progression. Despite improvements in intensive care management, including rapid cooling, hemodynamic optimization, and organ support, no specific pharmacological therapy targeting HS-ALI is currently available. Therefore, early identification of patients at risk and development of mechanism-based interventions remain major unmet clinical needs (2).

Current evidence suggests that HS-ALI results from the interaction of multiple pathological processes, including direct thermal stress, systemic inflammation, oxidative imbalance, endothelial injury, coagulation abnormalities, and microcirculatory disturbance (3) (**Figure 1**). Experimental studies indicate that excessive thermal stress can disrupt cellular protein homeostasis, impair mitochondrial function, and compromise hepatocyte integrity. However, the extent to which these experimental observations directly represent human HS-ALI remains incompletely defined. In particular, several proposed inflammatory mechanisms, including endotoxin translocation, Toll-like receptor signaling, and macrophage activation, are supported partly by evidence from sepsis and other inflammatory liver injury models rather than HS-specific clinical studies. Therefore, these pathways should currently be considered potential contributors rather than fully established mechanisms of HS-ALI (3). Increasing attention has been directed toward the role of cellular stress responses and programmed cell death (PCD) pathways in HS-associated tissue injury. Beyond apoptosis, emerging evidence suggests that pyroptosis, ferroptosis, necroptosis, and autophagy-related responses may participate in the regulation of inflammation, oxidative damage, and hepatocyte fate under severe stress conditions. Nevertheless, most mechanistic evidence regarding these pathways originates from experimental liver injury models, including ischemia–reperfusion injury, toxin-induced liver injury, and inflammatory disease models, rather than directly from HS-ALI. Consequently, the temporal dynamics, relative contribution, and clinical relevance of individual PCD pathways in HS-ALI remain largely unresolved. Similarly, potential biomarkers such as GSDMD-derived fragments or GPX4-related ferroptotic signatures require HS-specific validation before they can be considered reliable diagnostic or therapeutic targets.

**Figure 1:**
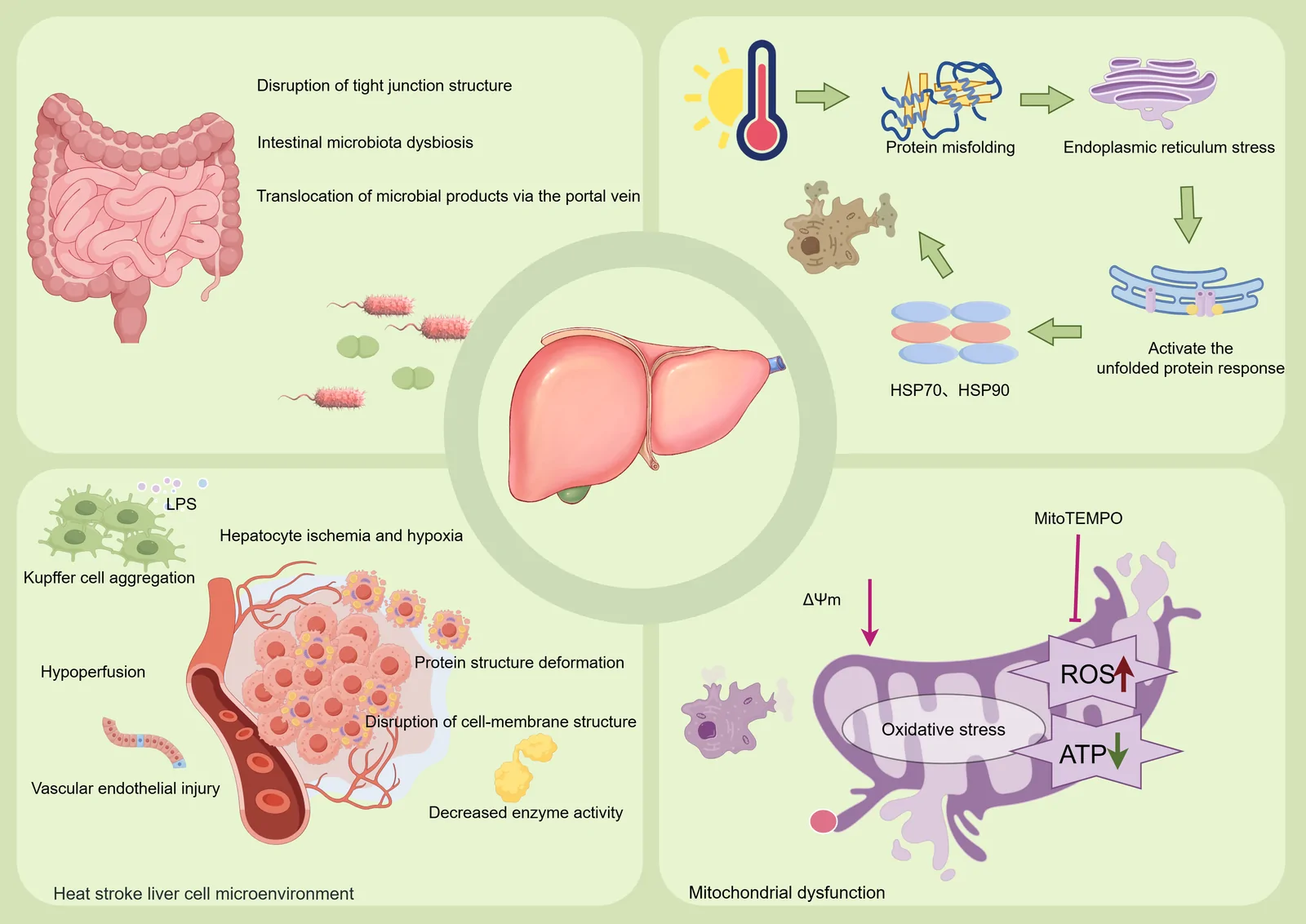
Overview of the major pathological mechanisms contributing to heat stroke-associated acute liver injury. Schematic summary of interconnected pathways by which heat stress drives organ injury. Barrier failure and microbial translocation (upper left): heat exposure disrupts epithelial tight junctions, promotes dysbiosis, and increases passage of microbial products (e.g., lipopolysaccharide, LPS) into the circulation (portal route shown as a representative axis). Microcirculatory dysfunction and ischemia–hypoxia (lower left): endothelial injury, hypoperfusion, and immune-cell activation (including Kupffer cell aggregation) establish an ischemic/hypoxic microenvironment that impairs hepatocellular structure and enzymatic function. Proteostasis collapse (upper right): heat-induced protein misfolding triggers endoplasmic reticulum (ER) stress and activates heat shock proteins (HSP70/HSP90) and the unfolded protein response (UPR). Mitochondrial stress and bioenergetic failure (lower right): defective mitophagy and loss of mitochondrial membrane potential (ΔΨm) amplify oxidative stress, characterized by increased reactive oxygen species (ROS), ATP depletion, and redox imbalance; MitoTEMPO is depicted as a representative mitochondrial ROS scavenger. Together, these coupled processes converge to propagate cellular damage and systemic dysfunction during heat stroke. LPS, lipopolysaccharide; ER, endoplasmic reticulum; HSP, heat shock protein; UPR, unfolded protein response; ROS, reactive oxygen species; ΔΨm, mitochondrial membrane potential.

Beyond its metabolic functions, the liver is an important immunological interface in which hepatocytes, Kupffer cells, liver sinusoidal endothelial cells, stellate cells, and infiltrating immune populations interact dynamically (4–6). Previous studies in inflammatory and ischemic liver diseases have demonstrated that immune-cell communication and inflammatory amplification contribute substantially to tissue damage and repair processes. In HS, emerging evidence suggests that systemic inflammation and disruption of the gut–liver axis may contribute to disease progression; however, whether these processes represent initiating events or secondary consequences of tissue injury remains uncertain (2, 3). Furthermore, age-related differences in thermoregulation, cardiovascular reserve, hydration homeostasis, and host susceptibility may influence the clinical presentation and outcome of HS (2). Although direct evidence defining age-specific mechanisms of HS-ALI remains limited, the potential implications for elderly and pediatric populations are discussed below.

Given the rapid progression and high mortality associated with HS-ALI, a comprehensive and balanced understanding of its pathogenesis is essential (3, 7). This review summarizes current knowledge regarding the epidemiology, clinical manifestations, molecular mechanisms, immune regulation, biomarkers, and therapeutic strategies of HS-ALI. Importantly, rather than considering mechanisms identified in other inflammatory diseases as definitive explanations for HS-ALI, we emphasize the distinction between direct HS evidence and extrapolated findings from related disease models. Throughout this review, we therefore distinguish clinical observations in patients with HS from HS-specific experimental evidence and from indirect mechanistic evidence extrapolated from other liver injury or inflammatory disease models. When evaluating individual mechanisms, greater weight is given to findings supported by human HS observations or HS-specific experimental models, whereas evidence derived solely from unrelated inflammatory or liver injury models is interpreted primarily as mechanistic plausibility rather than direct validation. By integrating clinical observations with experimental studies, this review aims to clarify current knowledge gaps and highlight future directions toward improved risk stratification, biomarker discovery, and targeted therapeutic development. Accordingly, the evidence discussed in this review can be broadly interpreted at three levels. Clinical evidence includes observations directly obtained from patients with heat stroke, such as patterns of liver dysfunction, coagulopathy, acute liver failure, treatment outcomes, and liver transplantation. HS-specific experimental evidence provides mechanistic support for selected pathways, including pyroptosis and ferroptosis, but does not by itself establish clinical efficacy or biomarker validity. Findings derived from sepsis, ischemia–reperfusion injury, drug-induced liver injury, or other non-HS models are used primarily to provide mechanistic context and are explicitly distinguished from direct HS evidence. Representative examples are summarized according to evidence level in **Table 1**.

**Table 1:** Evidence levels of representative biomarkers and therapeutic/interventional strategies in heat stroke-associated acute liver injury.

Category	Candidate/strategy	HS-specific evidence	Evidence from other settings	Current interpretation
Clinical biomarker	ALT, AST, bilirubin, PT/INR	Human clinical evidence; routinely monitored in HS-associated ALI/ALF and reflect hepatocellular injury or impaired synthetic function (7, 24)	Established in acute liver injury/failure of multiple etiologies	Clinically established indicators, but not specific for HS-ALI
Candidate biomarker	Circulating miR-122	No validated HS-ALI-specific clinical evidence	Human studies in other forms of acute liver injury and experimental hepatic I/R support its sensitivity to hepatocellular injury (159, 160)	Exploratory biomarker requiring prospective HS-specific validation
Candidate biomarker	GSDMD-N	HS-specific experimental evidence supports activation of the NLRP3–caspase-1–GSDMD pyroptosis pathway, but no validated human HS-ALI biomarker data are available (60, 64)	Additional experimental evidence supports GSDMD-dependent hepatocyte injury in other inflammatory liver diseases	Experimental mechanistic indicator; not a validated HS-ALI biomarker
Candidate biomarker/mechanistic target	GPX4 and ferroptosis-related signatures	HS-specific experimental studies support ferroptosis as a contributor to liver injury, but GPX4 has not been clinically validated as a biomarker (58, 59)	Extensive experimental evidence links GPX4-dependent ferroptosis to other forms of liver injury, including hepatic I/R	Experimental mechanistic indicator/target; clinical biomarker value unvalidated
Established clinical intervention	Rapid active cooling	Direct clinical/guideline evidence in HS	—	Established cornerstone of HS treatment; active cooling is recommended, with cold- or ice-water immersion achieving the fastest cooling when feasible (121)
Clinically used, HS-specific efficacy uncertain	N-acetylcysteine (NAC)	Used in small HS-ALI/ALF clinical series, but efficacy cannot be determined (7)	Randomized clinical evidence supports benefit in selected early-stage non-acetaminophen ALF (159)	Reasonable ALF-directed therapy in selected settings, but not an established HS-specific treatment
Extracorporeal support	High-volume plasma exchange (HVP/PE)	HS-specific evidence limited mainly to case-level experience	Randomized clinical trial in ALF demonstrated improved transplant-free survival and attenuation of systemic inflammation (147)	Supportive/bridging therapy; HS-specific efficacy remains uncertain
Extracorporeal support	MARS	No robust HS-specific trial evidence	Randomized ALF trial did not establish a clear survival advantage (151)	Supportive/bridging modality rather than definitive HS-ALF therapy
Organ support	CRRT	Used clinically when severe HS is complicated by AKI, metabolic disturbances, or other conventional indications	In ALF, observational data associate CRRT with greater ammonia reduction and improved short-term transplant-free outcomes (152)	Established renal/metabolic support; direct hepatoprotective efficacy in HS-ALI is unproven
Rescue therapy	Liver transplantation	HS-specific human clinical evidence, including multicenter retrospective data (24)	Established rescue therapy for irreversible ALF of selected etiologies	Definitive rescue option for carefully selected patients with non-recovering HS-ALF
Experimental therapeutic target	Ferroptosis inhibition (eg, liproxstatin-1, ferrostatin-1)	HS-specific animal evidence supports hepatoprotection with ferroptosis inhibition (58)	Additional preclinical evidence from hepatic I/R models (161, 162)	Preclinical; no established human HS-ALI efficacy
Experimental therapeutic target	Inflammasome/cytokine-directed therapy (NLRP3, IL-1, TNF-α, IL-6 pathways)	No established clinical efficacy in HS-ALI	Mechanistic and therapeutic evidence largely derived from other inflammatory diseases and experimental injury models	Experimental/hypothesis-generating
Experimental therapeutic strategy	Gut–liver axis and microbiota-directed interventions	No established HS-specific clinical benefit	Biological and clinical evidence derives mainly from critical illness and other gut–liver disorders	Exploratory; routine microbiome-targeted therapy is not currently supported

HS-ALI, heat stroke-associated acute liver injury; HS-ALF, heat stroke-associated acute liver failure; ALT, alanine aminotransferase; AST, aspartate aminotransferase; PT, prothrombin time; INR, international normalized ratio; GSDMD-N, N-terminal fragment of gasdermin D; GPX4, glutathione peroxidase 4; NAC, N-acetylcysteine; PE, plasma exchange; HVP, high-volume plasma exchange; MARS, Molecular Adsorbent Recirculating System; CRRT, continuous renal replacement therapy; ALF, acute liver failure; I/R, ischemia–reperfusion.

## 2 Epidemiological and clinical characteristics of heat stroke-associated liver injury

Heat stroke (HS) represents the most severe form of heat-related illness and is characterized by a combination of severe hyperthermia, central nervous system dysfunction, and systemic inflammatory responses, frequently accompanied by multiple organ dysfunction syndrome (MODS) (8, 9).With increasing global temperatures and more frequent extreme heat events, HS has become an important public health concern (10, 11). However, accurately estimating its epidemiological burden remains challenging because reported incidence varies substantially according to geographic region, climate conditions, population characteristics, and diagnostic criteria (12–14). Epidemiological studies have consistently demonstrated that heat waves, advanced age, chronic comorbidities, occupational heat exposure, and strenuous physical activity are major risk factors associated with severe HS and mortality (12–14). Clinically, HS can be broadly categorized into classic heat stroke (CHS) and exertional heat stroke (EHS). CHS typically occurs during environmental heat exposure and predominantly affects elderly individuals, patients with cardiovascular or neurological diseases, and those with impaired thermoregulatory capacity (10, 15). In contrast, EHS commonly develops in younger individuals, including athletes and military personnel, during intense physical activity under hot conditions (11, 13, 14). Although both forms share common pathological features, differences in triggering conditions, inflammatory responses, metabolic stress, and organ injury patterns suggest that HS-associated organ damage may exhibit considerable clinical heterogeneity (2, 16). Older adults and children represent clinically important vulnerable populations, although the biological basis of susceptibility differs across age groups (17–19). In older adults, impaired heat dissipation, reduced cardiovascular reserve, chronic comorbidities, and medication use may increase vulnerability to severe HS, whereas pediatric susceptibility is influenced by developmental physiology, environmental exposure, hydration status, and dependence on caregivers for recognition and heat avoidance (10, 12). Age-specific characteristics of HS-associated liver injury (20), however, remain poorly defined.

### 2.1 Differences in hepatic involvement between exertional and classic heat stroke

Although EHS and CHS share many core pathophysiological features, the clinical context in which hepatic injury develops differs substantially between the two phenotypes. Severe HS-associated acute liver injury and acute liver failure requiring transplantation have been reported predominantly in EHS cohorts (21, 22). In the Acute Liver Failure Study Group series, most affected patients were previously healthy young individuals exposed to excessive exertion, and hepatic injury occurred together with rhabdomyolysis, lactic acidosis, and acute kidney injury, indicating a broader syndrome of intense metabolic and multiorgan stress (7, 23). Similarly, the largest multicenter study specifically addressing severe HS-associated liver injury included 24 male patients with EHS and demonstrated that a small subset developed persistent profound coagulopathy and multiorgan dysfunction requiring liver transplantation (24). In contrast, CHS typically occurs in older individuals with impaired thermoregulatory capacity, chronic comorbidities, medication-related susceptibility, and reduced cardiovascular or renal reserve. Historical clinical cohorts of CHS reported that overt hepatic failure was relatively uncommon, despite substantial systemic morbidity, whereas rhabdomyolysis, acute renal failure, disseminated intravascular coagulation, and marked lactic acidosis were more characteristic of exertional than classic heat stroke (25, 26). These observations suggest that hepatic injury in EHS often develops in the setting of greater exertional and metabolic stress, whereas the clinical severity of CHS may be strongly influenced by age, comorbid disease, impaired physiological reserve, and delayed recognition. However, these differences should not be interpreted as evidence that EHS and CHS have fundamentally distinct liver-specific mechanisms. Direct head-to-head studies of hepatic injury are scarce, and recent reviews emphasize substantial overlap in the underlying pathophysiology of the two forms of HS (2, 16). Therefore, current evidence supports differences in clinical context and associated organ-injury patterns rather than a definitive distinction in HS-ALI pathogenesis.

### 2.2 Age-related susceptibility and clinical outcomes

Age may modify the risk and clinical outcomes of HS-associated organ injury primarily through differences in host susceptibility and physiological reserve; however, a distinct age-specific hepatic molecular pathway has not been established. Older adults exhibit reduced sweating and cutaneous vasodilation, attenuated cardiovascular responses, and impaired maintenance of hydration during heat exposure, all of which can limit heat dissipation and increase susceptibility to circulatory instability. These age-related alterations may theoretically aggravate hepatic hypoperfusion and systemic stress during HS, although direct liver-specific mechanistic evidence remains limited (17). Clinical outcome data further underscore the vulnerability of older populations, particularly in classic non-exertional heat stroke. In a multicenter cohort of 1,456 patients with non-exertional heat stroke, the mean age was 79 years, and age >80 years was independently associated with reduced 1-year survival, together with factors including diuretic use, cardiac disease, hypotension, and severe hyperthermia (15). Similarly, a French ICU cohort demonstrated high mortality in severe heat stroke and identified prolonged prothrombin time, high illness-severity scores, and hemodynamic instability among predictors of death (27). These studies support age-associated vulnerability at the systemic level, but they do not establish that aging independently worsens liver-specific outcomes once HS-ALI has developed.

Pediatric HS represents a less well-studied but clinically important setting. Current pediatric consensus recognizes children as a population susceptible to heat stroke and associated organ injury and emphasizes the importance of rapid recognition, cooling, fluid management, and monitoring for multiorgan complications (28). However, evidence specifically addressing HS-ALI in children is sparse. Pediatric acute liver failure caused by HS has been reported, including a recent case of a previously healthy 9-year-old child who developed hepatic encephalopathy, severe coagulopathy, and marked aminotransferase elevation following exertional heat stroke and subsequently recovered with supportive management including N-acetylcysteine (20). Such reports confirm that severe hepatic involvement can occur in children but are insufficient to determine whether pediatric patients have a distinct susceptibility, molecular mechanism, or prognosis compared with adults. Overall, current evidence indicates that age strongly modifies susceptibility to severe heat stroke and systemic outcomes, whereas whether age independently alters the molecular pathogenesis, severity, or regenerative capacity of HS-ALI remains unknown. Future multicenter studies should therefore incorporate age-stratified analyses of hepatic injury trajectories, coagulation abnormalities, inflammatory responses, and recovery.

### 2.3 Heat stroke-associated acute liver injury

Among the organs affected by HS, the liver is particularly vulnerable and is an important determinant of disease severity and prognosis. Across both HS phenotypes, clinically significant hepatic involvement may progress rapidly from hepatocellular injury to severe coagulopathy and acute liver failure, underscoring the importance of dynamic hepatic monitoring during the early disease course (7, 21, 22). Typical laboratory abnormalities include dramatic increases in alanine aminotransferase (ALT) and aspartate aminotransferase (AST), which may reach several thousand units per liter, together with progressive impairment of hepatic synthetic function reflected by prolonged prothrombin time (PT), elevated international normalized ratio (INR), hyperbilirubinemia, and, in severe cases, hepatic encephalopathy (7, 22, 23).

A major clinical challenge in HS-ALI is the rapid and heterogeneous disease trajectory. Unlike many conventional forms of acute liver injury, hepatic damage in HS may continue to progress despite successful temperature reduction and initial supportive management, suggesting that secondary biological processes may contribute to ongoing injury after the initial thermal insult (29). Based on clinical observations from case reports and cohort studies, HS-ALI may present with different temporal patterns, including early-onset liver injury occurring within the first 24–48 hours and delayed progression developing several days after heat exposure (21, 22). However, these patterns have not yet been validated as formal clinical classifications and should be considered descriptive phenotypes rather than established disease subtypes. Current clinical assessment of HS-ALI primarily relies on conventional biochemical indicators, including ALT, AST, bilirubin, and coagulation parameters (21, 22). Although these markers are useful for evaluating hepatocellular injury and predicting disease severity, they often reflect relatively late-stage damage and provide limited information regarding early risk prediction or mechanistic stratification (30, 31). Recently, systemic scoring systems such as the Sequential Organ Failure Assessment (SOFA) score have been increasingly applied to evaluate MODS severity in critically ill patients, including those with HS (32, 33). In addition, emerging biomarkers, including circulating microRNAs and immune-related molecules, have shown potential value in early detection of liver injury in experimental and clinical settings; however, their specific diagnostic and prognostic roles in HS-ALI require further validation in larger prospective cohorts (30, 34).

Overall, HS-associated liver injury represents a rapidly progressive and clinically heterogeneous complication of severe heat exposure Current evidence supports its association with substantial morbidity and mortality, but significant knowledge gaps remain regarding epidemiological characteristics, age-related susceptibility, early diagnostic markers, and validated risk stratification strategies. Future studies integrating clinical phenotyping, longitudinal biomarker monitoring, and mechanistic investigation are required to establish evidence-based approaches for early recognition and targeted management of HS-ALI.

## 3 Pathophysiological mechanisms of heat stroke-associated liver injury

### 3.1 Direct cytotoxicity of hyperthermia

Hyperthermia represents one of the earliest and most fundamental stressors during heat stroke (HS). Experimental and clinical observations indicate that excessive elevation of core temperature can directly impair cellular homeostasis through protein denaturation, membrane instability, mitochondrial dysfunction, and disruption of intracellular signaling networks (8, 9).The liver is considered particularly susceptible to thermal stress because of its high metabolic activity, abundant mitochondrial content, and central role in systemic metabolism and detoxification. However, the precise contribution of direct thermal cytotoxicity to human HS-associated acute liver injury (HS-ALI) remains difficult to isolate clinically because thermal damage occurs simultaneously with circulatory disturbance, inflammation, and immune activation (3, 35). 

Early experimental studies demonstrated that severe hyperthermia can induce structural abnormalities in hepatocytes, including mitochondrial swelling, endoplasmic reticulum disruption, and loss of cellular integrity (36). At temperatures exceeding physiological tolerance, heat stress may promote protein misfolding, aggregation of damaged proteins, and impairment of enzymatic activity, ultimately compromising hepatocyte function (37). These observations provide a biological basis for direct thermal injury; however, most mechanistic evidence originates from experimental heat exposure models rather than human HS-ALI studies (3, 35, 38). In addition to direct thermal effects, heat exposure frequently occurs together with systemic circulatory stress. During HS, redistribution of blood flow toward the skin to facilitate heat dissipation, combined with cardiovascular dysfunction under prolonged thermal stress, may reduce visceral perfusion and contribute to hepatic hypoxia (9). Experimental and clinical studies suggest that microcirculatory impairment, endothelial activation, and relative ischemia may participate in HS-associated organ dysfunction (35, 39). Nevertheless, whether hepatic injury results primarily from direct heat toxicity or secondary ischemic-like injury remains unresolved. The concept of “ischemia–reperfusion-like injury” has been proposed based on similarities between HS and other systemic inflammatory states, but direct evidence specifically demonstrating this process in HS-ALI remains limited (3, 35). 

Mitochondria are considered central targets of heat-induced cellular stress. Hyperthermia can disrupt mitochondrial membrane potential, impair oxidative phosphorylation, and increase reactive oxygen species (ROS) generation in experimental models (35, 38). Excessive ROS accumulation may promote lipid peroxidation, DNA damage, and activation of cell death pathways (38). However, mitochondrial dysfunction in HS should be interpreted within the broader context of systemic oxidative stress, as similar mitochondrial abnormalities have also been observed in sepsis, ischemia–reperfusion injury, and other forms of acute liver injury. Therefore, although mitochondrial injury represents a plausible mechanism linking thermal stress to hepatocellular dysfunction, its specific contribution to HS-ALI requires further investigation (3, 35). 

Heat shock proteins (HSPs), particularly HSP70 and HSP90 represent an important adaptive response to hyperthermia. These molecular chaperones maintain protein stability, facilitate refolding or degradation of damaged proteins, and participate in cellular stress tolerance (37). Experimental studies have shown that HSP70 induction can exert cytoprotective effects by limiting oxidative stress and inflammatory signaling during heat exposure (40). However, excessive or prolonged heat stress may overwhelm these protective mechanisms, resulting in accumulation of damaged proteins and loss of cellular homeostasis. Thus, HSP activation should not be considered solely as a pathogenic process but rather as a dynamic compensatory response reflecting the balance between cellular adaptation and injury (3, 35). Heat stress may also affect extrahepatic systems that indirectly influence liver injury For example, disruption of intestinal barrier integrity during HS has been reported to facilitate translocation of microbial products, potentially amplifying systemic inflammation and hepatic immune activation. However, similar to endotoxin-related pathways, the direct causal relationship between intestinal barrier disruption and HS-ALI requires further validation in human studies (3, 35). Taken together, direct hyperthermic cytotoxicity is an important initiating component of HS-associated liver injury, involving protein instability, mitochondrial stress, oxidative imbalance, and impaired cellular adaptation (3, 35, 38). Nevertheless, current evidence suggests that direct thermal injury alone is unlikely to fully explain the rapid progression of HS-ALI Instead, hyperthermia-induced cellular stress likely interacts with subsequent inflammatory responses, microcirculatory dysfunction, immune activation, and regulated cell death pathways to determine disease severity (3, 35). Future HS-specific studies integrating temporal analyses and clinical samples are required to clarify the relative contribution of direct thermal injury within the complex pathophysiological network of HS-ALI. 

### 3.2 Gastrointestinal inflammation and the LPS-TLR4 pathway

Beyond direct thermal injury, increasing evidence suggests that gastrointestinal dysfunction may represent an important component of systemic inflammatory responses during heat stroke (HS). The intestine is highly sensitive to thermal stress because of its high metabolic demand and dependence on adequate microvascular perfusion. Experimental studies have demonstrated that heat exposure can impair intestinal barrier integrity by disrupting epithelial tight junction proteins, increasing intestinal permeability, and promoting mucosal injury (41). These alterations may facilitate the translocation of microbial components, including lipopolysaccharide (LPS) derived from Gram-negative bacteria, into the circulation (35, 42). 

The concept of the gut–liver axis provides a potential framework for understanding how intestinal disturbances may influence hepatic injury during systemic stress. Anatomically, the liver receives blood directly from the portal circulation and continuously encounters microbial products originating from the intestine Under physiological conditions, Kupffer cells and other hepatic immune components maintain immune surveillance by clearing microbial molecules and regulating inflammatory responses. However, excessive exposure to pathogen-associated molecular patterns (PAMPs), including LPS, may induce hepatic immune activation under pathological conditions.

The LPS–Toll-like receptor 4 (TLR4) signaling pathway has been extensively characterized in sepsis and other inflammatory liver diseases. LPS binding to TLR4 activates MyD88-dependent and TRIF-dependent signaling cascades, leading to activation of transcription factors such as nuclear factor-κB (NF-κB) and subsequent production of inflammatory mediators including TNF-α, IL-1β, and IL-6 (43, 44).Activation of Kupffer cells through TLR4 signaling has been implicated in various forms of liver injury, including ischemia–reperfusion injury, alcoholic liver disease, and toxin-induced liver damage (45, 46). However, it is important to emphasize that direct evidence demonstrating that LPS–TLR4 signaling is a dominant driver of HS-associated acute liver injury (HS-ALI) remains limited Current interpretations largely rely on mechanistic similarities between HS and other systemic inflammatory disorders, particularly sepsis (3, 35, 47).

Several experimental observations support the possibility that intestinal dysfunction contributes to HS-induced systemic inflammation Heat stress can impair intestinal epithelial integrity, increase circulating endotoxin levels, and promote inflammatory responses in experimental models. Moreover, elevated circulating endotoxin and inflammatory mediators have been observed in severe heat stroke patients, suggesting that microbial translocation may participate in disease progression (42). Nevertheless, whether endotoxin translocation represents an initiating event, an amplifier of systemic inflammation, or a secondary consequence of intestinal ischemia during HS remains unresolved (35). Following potential endotoxin exposure, activated hepatic macrophages may release inflammatory mediators and reactive oxygen species (ROS), thereby contributing to local oxidative stress and endothelial dysfunction. Similar macrophage-mediated inflammatory amplification has been extensively described in sepsis-associated liver injury and ischemia–reperfusion models. However, extrapolation of these mechanisms to HS-ALI requires caution because the magnitude, timing, and cellular sources of inflammatory activation may differ substantially between HS and infectious or ischemic conditions.

Recent studies have broadened our understanding of the gut–liver axis beyond simple endotoxin translocation, highlighting complex interactions among microbial metabolites, epithelial integrity, innate immune cells, and systemic inflammatory networks. In HS, emerging evidence suggests that intestinal barrier disruption and altered immune homeostasis may contribute to disease progression; however, the precise molecular pathways and their clinical significance remain insufficiently characterized (2, 35) Future studies integrating longitudinal clinical sampling, microbiome profiling, and HS-specific experimental models are required to determine whether gut-derived signals represent actionable therapeutic targets in HS-ALI. Collectively, gastrointestinal inflammation and LPS-mediated immune activation represent a plausible but incompletely validated mechanism linking systemic heat stress to hepatic injury. Current evidence supports the involvement of intestinal barrier dysfunction and innate immune activation in HS, while the specific contribution of the LPS–TLR4 pathway to HS-ALI requires further direct investigation (3, 35, 47).

### 3.3 Oxidative stress and mitochondrial dysfunction

Oxidative imbalance and mitochondrial dysfunction have been proposed as important contributors to heat stroke-associated acute liver injury (HS-ALI) (3, 35, 38). The liver has a high metabolic demand and relies extensively on mitochondrial oxidative phosphorylation to maintain cellular energy homeostasis. During heat stroke (HS), excessive thermal stress, systemic inflammation, and circulatory abnormalities may collectively disturb mitochondrial function and redox balance. However, the relative contribution of primary thermal mitochondrial injury versus secondary inflammatory and ischemic stress remains incompletely defined, particularly in human HS-ALI. Experimental studies indicate that hyperthermia can impair mitochondrial integrity by disrupting electron transport chain activity, reducing mitochondrial membrane potential, and increasing reactive oxygen species (ROS) generation (35, 38). Excessive ROS accumulation may subsequently damage mitochondrial DNA, membrane lipids, and proteins, resulting in impaired ATP production and cellular dysfunction (48). In addition, systemic circulatory stress during HS may further aggravate mitochondrial dysfunction by limiting oxygen availability and increasing the mismatch between energy demand and supply. Similar mitochondrial abnormalities have been extensively described in sepsis and ischemia–reperfusion injury, suggesting potential mechanistic overlap; however, whether these pathways occur with the same magnitude and temporal pattern in HS-ALI requires direct investigation.

ROS generation represents a critical link between mitochondrial stress and inflammatory activation. In multiple inflammatory disease models, excessive ROS production can activate redox-sensitive signaling pathways, including NF-κB and MAPK pathways (49), thereby promoting inflammatory cytokine production ROS-mediated activation of the NLRP3 inflammasome has also been widely reported in sterile inflammatory conditions and liver injury models (50). Nevertheless, direct evidence demonstrating that ROS-driven NLRP3 activation is a dominant mechanism in HS-ALI remains limited, and most available evidence originates from non-HS inflammatory models.

Mitochondrial stress responses are not exclusively detrimental Heat shock proteins and endogenous antioxidant systems, including glutathione (GSH), superoxide dismutase (SOD), and nuclear factor erythroid 2-related factor 2 (Nrf2)-dependent pathways, represent important adaptive mechanisms that maintain redox homeostasis under stress conditions (51). Experimental studies in other oxidative injury models have demonstrated that activation of Nrf2 signaling can reduce oxidative damage and preserve mitochondrial function (51). However, whether pharmacological modulation of mitochondrial antioxidant pathways provides therapeutic benefit in human HS-ALI remains unknown. Overall, oxidative stress and mitochondrial dysfunction likely represent important intermediate processes connecting thermal stress, metabolic disturbance, inflammation, and hepatocyte injury during HS (3, 35, 38). Nevertheless, current evidence remains largely derived from experimental models, and the precise temporal relationship between mitochondrial injury, inflammatory amplification, and programmed cell death in HS-ALI requires further clarification through HS-specific longitudinal studies.

### 3.4 Coagulation dysfunction and microcirculatory disturbance

Coagulation abnormalities represent a prominent clinical feature of severe heat stroke and contribute substantially to multiple organ dysfunction, including hepatic injury (39, 52). Clinical studies have demonstrated that HS patients frequently exhibit elevated D-dimer levels, prolonged coagulation times, thrombocytopenia, and laboratory features compatible with disseminated intravascular coagulation (DIC) in severe cases (27, 53). These abnormalities indicate that HS is not only a thermal injury syndrome but also a systemic vascular and hemostatic disorder (39, 52). The mechanisms underlying coagulation disturbance in HS are complex and involve interactions among endothelial injury, inflammation, platelet activation, and impaired hepatic synthetic function (54). Heat stress and systemic inflammatory responses may activate vascular endothelial cells and promote the release of endothelial-derived mediators, including von Willebrand factor (vWF), which contributes to platelet adhesion and microvascular thrombosis (39, 55, 56) (**Figure 2**). Similar endothelial activation patterns have been described in sepsis and other systemic inflammatory states, suggesting possible mechanistic similarities; however, the specific endothelial response profile in HS requires further characterization.

**Figure 2:**
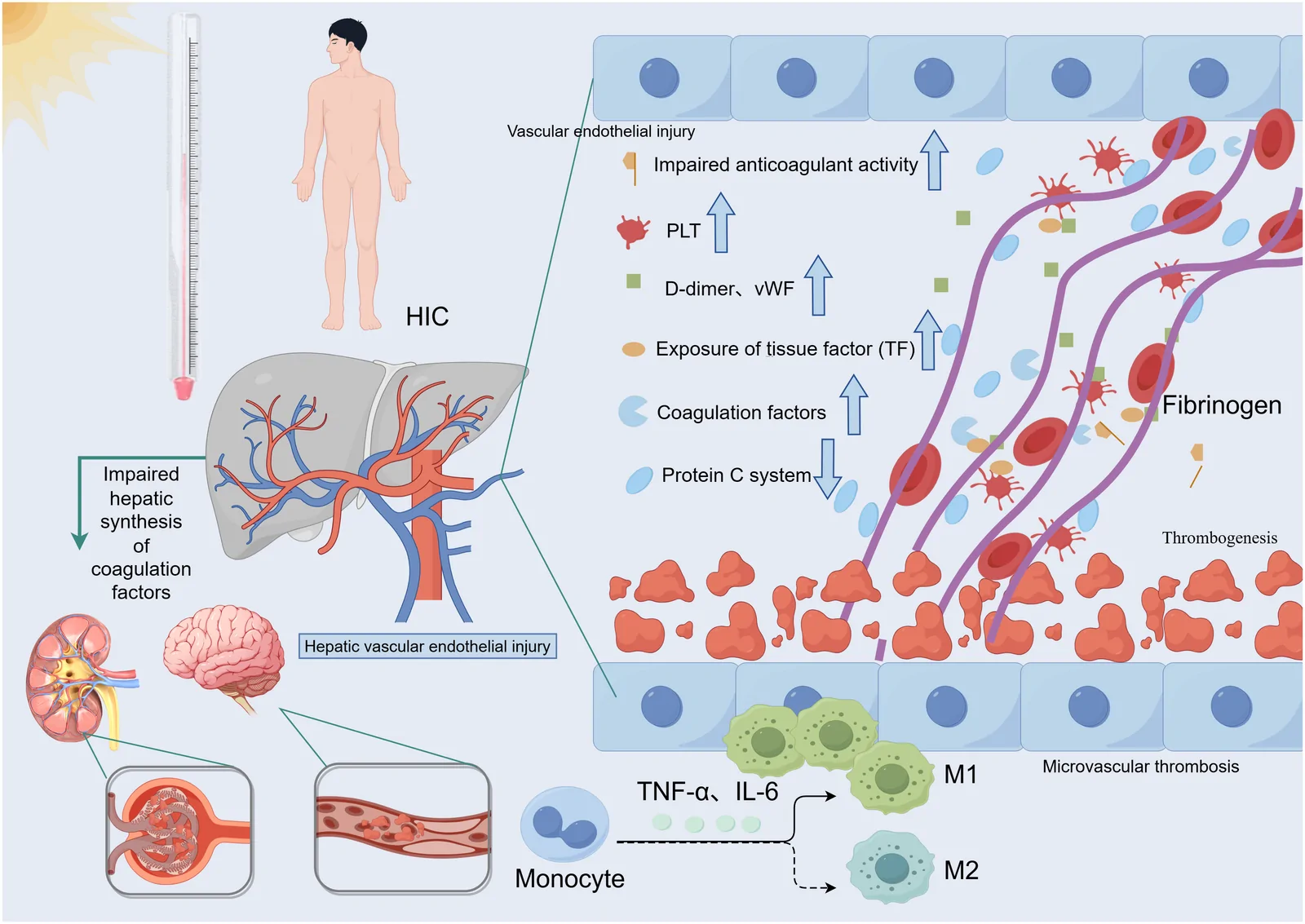
Heat stroke–induced coagulopathy and immunothrombotic multi-organ injury. Schematic overview of the interconnected coagulation–inflammation cascade in heat stroke. Heat stress provokes vascular endothelial activation/injury and perturbs hepatic homeostasis, promoting exposure of tissue factor (TF) and release of von Willebrand factor (vWF), together with platelet activation/aggregation and dysregulated coagulation factor availability (including impaired hepatic synthesis). Concurrently, attenuation of endogenous anticoagulant pathways (e.g., the protein C system) shifts the hemostatic balance toward thrombin generation and fibrin formation, leading to microvascular thrombosis and consumption coagulopathy; increased D-dimer is depicted as a representative readout of fibrin turnover. Microthrombi obstruct capillary networks, causing tissue hypoperfusion/ischemia and contributing to organ dysfunction (e.g., kidney and brain). In parallel, recruited monocytes polarize toward pro-inflammatory M1 macrophages that release cytokines (notably TNF-α and IL-6), further amplifying endothelial injury and coagulation activation, whereas M2 macrophages support inflammation resolution. Collectively, these processes drive heat stroke–induced coagulopathy (HIC) and multi-organ dysfunction. HS, heat stroke; HIC, heat stroke–induced coagulopathy; TF, tissue factor; vWF, von Willebrand factor; PLT, platelet; TNF-α, tumor necrosis factor-α; IL-6, interleukin-6.

The liver plays a central role in maintaining coagulation homeostasis because it synthesizes most circulating coagulation factors and anticoagulant proteins. During HS-associated liver dysfunction, impaired synthesis of coagulation factors, including fibrinogen and factor V, may contribute to progressive coagulopathy (35, 52). Importantly, HS-associated coagulation abnormalities may exhibit a dynamic transition from early hypercoagulability to later bleeding tendency, reflecting the imbalance between coagulation activation, consumption of clotting factors, and impaired hepatic synthetic capacity (39, 55). Microcirculatory dysfunction represents another potential mechanism linking coagulation abnormalities with hepatic injury. Excessive coagulation activation and endothelial dysfunction may promote microvascular obstruction, impair sinusoidal perfusion, and aggravate tissue hypoxia (39, 52). Similar pathological processes have been demonstrated in sepsis-associated microvascular injury; however, whether comparable microthrombotic changes directly occur in HS-ALI remains insufficiently characterized. Therefore, the concept of HS-induced “ischemia–reperfusion-like injury” should currently be interpreted as a proposed mechanism rather than an established pathway (3, 35).

Inflammatory activation may further amplify coagulation abnormalities through reciprocal interactions between immune cells and the vasculature (57). Cytokines such as TNF-α and IL-6 can enhance tissue factor expression and suppress physiological anticoagulant pathways, including the protein C system, contributing to immunothrombotic responses. However, most mechanistic insights into immunothrombosis originate from infection-associated conditions, and their specific relevance to HS-associated liver injury remains an important area for future investigation (39, 52).

In summary, coagulation dysfunction and microcirculatory disturbance are clinically important components of severe HS and may contribute to hepatic injury progression. Future studies integrating coagulation profiling, endothelial biomarkers, and longitudinal clinical monitoring are needed to determine whether coagulation-related pathways can serve as early predictors or therapeutic targets for HS-ALI.

## 4 The role of programmed cell death mechanisms in HS-ALI

Heat stroke (HS) induces a complex cellular stress environment characterized by hyperthermia, oxidative imbalance, mitochondrial dysfunction, inflammation, and metabolic disturbance. Under these conditions, hepatocytes and hepatic immune cells may engage multiple regulated cell death pathways, including pyroptosis, ferroptosis, and necroptosis, together with autophagy-related stress responses (35, 58–60). Increasing experimental evidence suggests that programmed cell death (PCD) pathways participate in acute liver injury; however, the precise contribution and temporal relationship of individual PCD pathways in heat stroke-associated acute liver injury (HS-ALI) remain incompletely understood.

Unlike classical apoptosis, several non-apoptotic cell death pathways have immunological consequences because they can promote the release of damage-associated molecular patterns (DAMPs) and amplify inflammatory responses (61, 62). Nevertheless, most mechanistic knowledge regarding these pathways originates from sepsis, ischemia–reperfusion injury, drug-induced liver injury, or metabolic liver disease models rather than HS-specific investigations. Therefore, PCD mechanisms discussed below should be interpreted as potential contributors to HS-ALI progression rather than fully validated disease-specific pathways (35).

### 4.1 Pyroptosis: a potential amplifier of inflammatory injury

Pyroptosis is an inflammatory form of programmed cell death characterized by inflammasome activation, caspase-1-dependent cleavage of gasdermin D (GSDMD), membrane pore formation, and release of inflammatory cytokines including IL-1β and IL-18 Compared with apoptosis, pyroptosis directly links cell death with innate immune activation and therefore has attracted increasing attention in inflammatory liver injury (62). In HS, several mechanisms may theoretically promote pyroptotic activation Severe hyperthermia can induce cellular stress, mitochondrial dysfunction, oxidative imbalance, and release of intracellular danger signals such as ATP and HMGB1 Meanwhile, heat stress-associated intestinal barrier disruption may increase exposure to microbial products, including LPS, which is capable of activating innate immune pathways. However, the majority of evidence supporting LPS/TLR4/NLRP3-mediated pyroptosis originates from sepsis or other inflammatory models, and whether this pathway represents a dominant mechanism in HS-ALI remains uncertain (35, 60).

Importantly, the biological relevance of pyroptosis to HS-associated liver injury is supported by HS-specific experimental evidence. In a rat HS model, hepatic inflammasome assembly and caspase-1 activation promoted IL-1β maturation and hepatocyte pyroptosis, contributing directly to liver injury (60). Subsequent work demonstrated that extracellular histones aggravate HS-induced liver injury through TLR9–NLRP3-dependent hepatocyte pyroptosis (63) (**Figure 3**). More recently, an HS-specific rat and hepatocyte study identified activation of the NLRP3–caspase-1–GSDMD axis, providing more direct experimental support for GSDMD-dependent pyroptotic signaling in this setting (64).Nevertheless, these studies provide mechanistic evidence rather than validation of circulating GSDMD-N as a diagnostic or prognostic biomarker in patients with HS-ALI. Although HS-specific experimental evidence now supports GSDMD-dependent pyroptotic signaling, the clinical relevance of GSDMD-N as a circulating biomarker has not been established. No adequately validated human HS-ALI study has yet demonstrated that circulating GSDMD-N provides diagnostic, prognostic, or treatment-response information beyond conventional clinical indicators. Therefore, GSDMD-N should currently be regarded as an experimental mechanistic readout rather than a validated HS-ALI biomarker. Thus, pyroptosis may represent an important inflammatory amplification mechanism during HS-associated liver injury, but current evidence supports its role as a candidate pathway rather than a confirmed therapeutic target (60, 63, 64).

**Figure 3:**
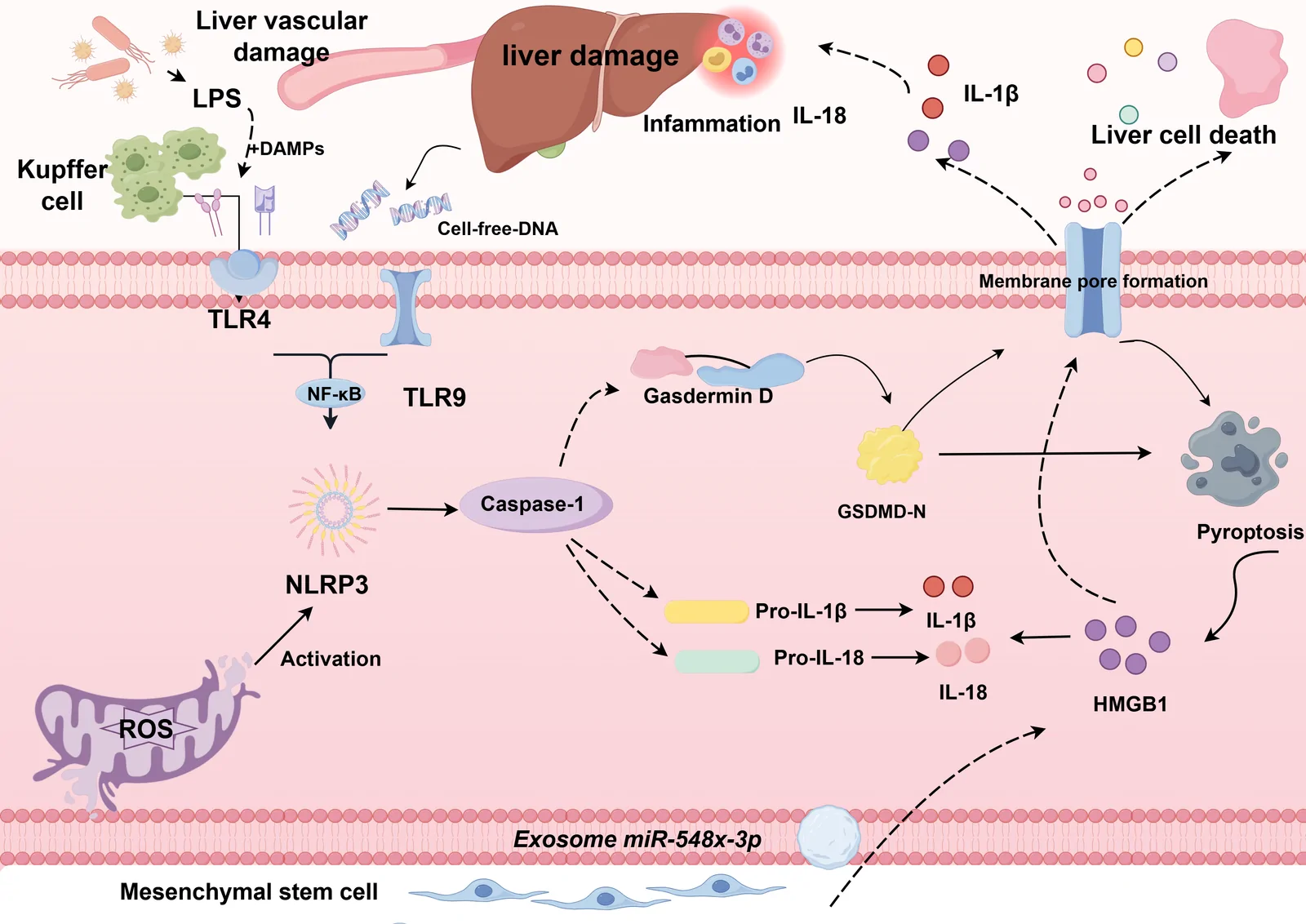
TLR4/TLR9–NLRP3 inflammasome activation drives pyroptosis and inflammatory amplification in liver injury, with modulation by MSC-derived exosomal miR-548x-3p. Schematic depiction of a pyroptosis-centered inflammatory cascade during liver injury. Hepatic vascular injury and cellular stress increase exposure to microbe-derived pathogen-associated molecular patterns (PAMPs) (e.g., LPS) and release of damage-associated molecular patterns (DAMPs) (e.g., HMGB1 and extracellular histones). These signals activate Kupffer cells and other hepatic innate immune cells through TLR4 and TLR9, providing priming and/or activation cues for NLRP3 inflammasome assembly. Inflammasome activation promotes caspase-1 cleavage, which (i) processes pro–IL-1β and pro–IL-18 into mature IL-1β and IL-18, and (ii) cleaves gasdermin D (GSDMD) to generate membrane pores, thereby triggering pyroptotic cell death and facilitating cytokine release. The resultant inflammatory amplification contributes to progressive hepatocellular injury and liver cell death. Mesenchymal stem cell (MSC)-derived exosomal miR-548x-3p is depicted as an inhibitory modulator of HMGB1-related pyroptotic signaling based on experimental evidence from heat-stressed vascular endothelial cells. Its direct role in hepatocyte pyroptosis during HS-ALI remains to be established. LPS, lipopolysaccharide; PAMPs, pathogen-associated molecular patterns; DAMPs, damage-associated molecular patterns; HMGB1, high mobility group box 1; TLR, toll-like receptor; NLRP3, NOD-like receptor family pyrin domain-containing 3; GSDMD, gasdermin D; IL, interleukin; MSC, mesenchymal stem cell.

### 4.2 Ferroptosis: a potential link between iron metabolism and oxidative damage

Ferroptosis is a regulated cell death pathway characterized by iron-dependent lipid peroxidation and failure of antioxidant defense systems (**Figure 4**). Since its discovery, ferroptosis has been implicated in various liver diseases, including ischemia–reperfusion injury, alcoholic liver disease, and drug-induced liver injury (65). Theoretically, HS creates several conditions favorable for ferroptotic activation, including excessive ROS production, mitochondrial dysfunction, disturbed iron metabolism, and depletion of antioxidant capacity (58, 59).The glutathione peroxidase 4 (GPX4)-glutathione system represents a major defense mechanism against lipid peroxidation Loss of GPX4 activity can lead to accumulation of lipid hydroperoxides and subsequent ferroptotic cell death (59). Although clinical evidence directly linking ferroptosis to HS-ALI remains limited, HS-specific experimental studies support its biological relevance in liver injury. In a mouse model of HS-ALI, ferroptosis-related oxidative injury was observed, and pharmacological inhibition with liproxstatin-1 attenuated hepatic injury, supporting a causal contribution of ferroptotic cell death (58, 59). In a separate HS-specific study, HMOX1-mediated ferroptosis of Kupffer cells, particularly a Clec4F^+^/CD206^+^ subset, was shown to promote NLRP3 activation and IL-1β-associated hepatic inflammation (59). These findings strengthen the biological relevance of ferroptosis to HS-ALI, although they do not establish GPX4 itself as a clinically validated biomarker.

**Figure 4:**
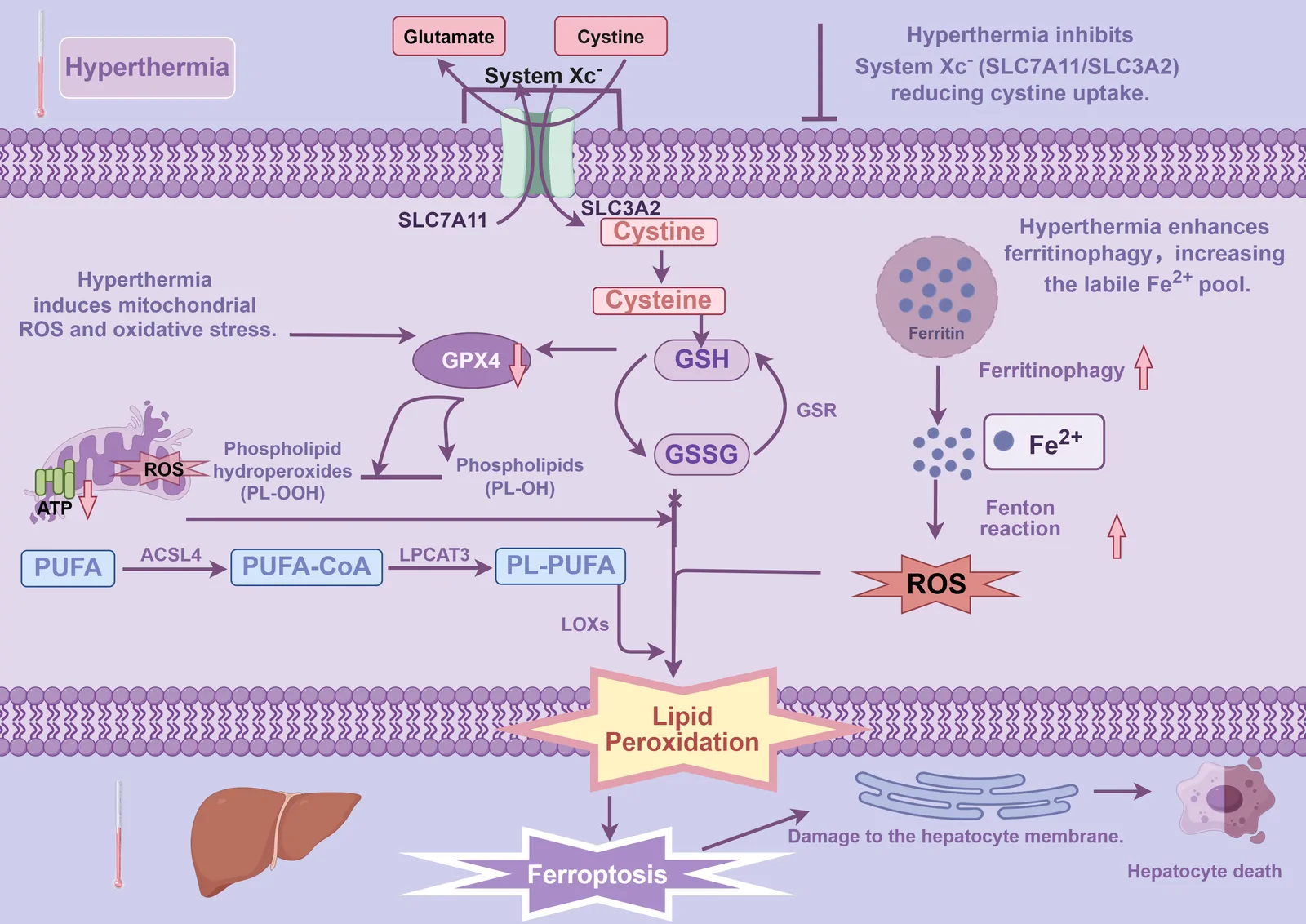
Proposed ferroptosis-related pathways contributing to hepatocyte injury during hyperthermia. Schematic overview of the lipid peroxidation cascade that underlies hyperthermia-induced ferroptosis in hepatocytes. Hyperthermia disrupts plasma membrane homeostasis and compromises the cystine/glutamate antiporter system Xc− (SLC7A11/SLC3A2), limiting cystine uptake and thereby reducing intracellular cysteine availability for glutathione (GSH) synthesis and redox cycling (GSH/GSSG, supported by GSR). Depletion of GSH diminishes GPX4 activity, weakening detoxification of phospholipid hydroperoxides and predisposing membranes to oxidative damage. In parallel, hyperthermia enhances PUFA remodeling into phospholipids (PUFA → PUFA-CoA → PL-PUFA, involving ACSL4, LPCAT3, and lipid-oxidizing LOXs), thereby increasing the abundance of oxidation-susceptible membrane substrates. Iron dysregulation further amplifies oxidative injury: elevation of labile Fe²^+^ and increased ferritinophagy expand the reactive iron pool, promoting Fenton chemistry and hydroxyl radical generation, which accelerates phospholipid peroxidation. Mitochondrial dysfunction (ROS accumulation and ATP decline) and inflammatory cues are depicted as additional amplifiers of redox imbalance. Collectively, these convergent events drive lipid peroxidation, execute ferroptosis, and culminate in hepatocyte death. system Xc−, cystine/glutamate antiporter; SLC7A11/SLC3A2, system Xc− subunits; GSH/GSSG, reduced/oxidized glutathione; GSR, glutathione reductase; GPX4, glutathione peroxidase 4; PUFA, polyunsaturated fatty acid; PL-PUFA, PUFA-containing phospholipids; ACSL4, acyl-CoA synthetase long-chain family member 4; LPCAT3, lysophosphatidylcholine acyltransferase 3; LOXs, lipoxygenases; Fe²^+^, ferrous iron.

The relationship between ferroptosis and inflammation is particularly relevant in HS Lipid peroxidation products generated during ferroptosis can act as endogenous inflammatory signals and potentially modulate macrophage activation (59, 65). However, whether ferroptotic hepatocytes directly regulate immune responses in HS-ALI remains unknown. Furthermore, ferritinophagy, an autophagy-dependent process involving ferritin degradation and iron release, has been proposed as a regulator of ferroptosis (66). Nevertheless, the interaction between autophagy and ferroptosis in HS-ALI has not been systematically characterized. Although HS-specific experimental evidence supports ferroptosis as a biologically relevant pathway, GPX4 should still be considered a mechanistic indicator rather than a validated clinical biomarker for HS-ALI. Its diagnostic and prognostic performance has not been established in well-characterized human HS cohorts Whether ferroptosis precedes, follows, or occurs concurrently with other regulated cell death pathways during HS-ALI remains unknown and requires dedicated longitudinal studies.

### 4.3 Necroptosis: a potential inflammatory cell death pathway requiring validation in HS-ALI

Necroptosis is a form of regulated necrotic cell death characterized by loss of plasma membrane integrity and release of intracellular components, including damage-associated molecular patterns (DAMPs), which can amplify inflammatory responses. Unlike apoptosis, necroptosis results in cellular membrane rupture and therefore has strong immunological consequences (61).The canonical necroptotic pathway is primarily regulated by receptor-interacting protein kinase 1 (RIPK1), receptor-interacting protein kinase 3 (RIPK3), and mixed lineage kinase domain-like protein (MLKL). Upon appropriate stimulation, phosphorylated RIPK3 activates MLKL, leading to membrane disruption and inflammatory cell death (67). In liver diseases, necroptosis has been implicated in several pathological conditions, including ischemia–reperfusion injury, viral hepatitis, and toxin-induced liver injury. Experimental studies suggest that excessive inflammatory signaling, mitochondrial stress, and impaired apoptotic regulation may promote necroptotic activation under severe cellular stress conditions (67).These findings provide a biological rationale for considering necroptosis as a potential contributor to HS-ALI. However, direct evidence demonstrating a critical role of necroptosis in HS-associated liver injury remains limited. Heat stroke induces multiple overlapping stress responses, including hyperthermia, oxidative injury, inflammation, and metabolic disturbance, all of which may theoretically influence RIPK1–RIPK3–MLKL signaling (68) (**Figure 5**). Nevertheless, most mechanistic evidence regarding necroptosis-mediated liver injury originates from non-HS models, particularly ischemia–reperfusion and inflammatory liver diseases. Therefore, whether necroptosis represents a primary driver, a secondary consequence of irreversible hepatocyte damage, or an amplification mechanism during HS-ALI remains unclear.

**Figure 5:**
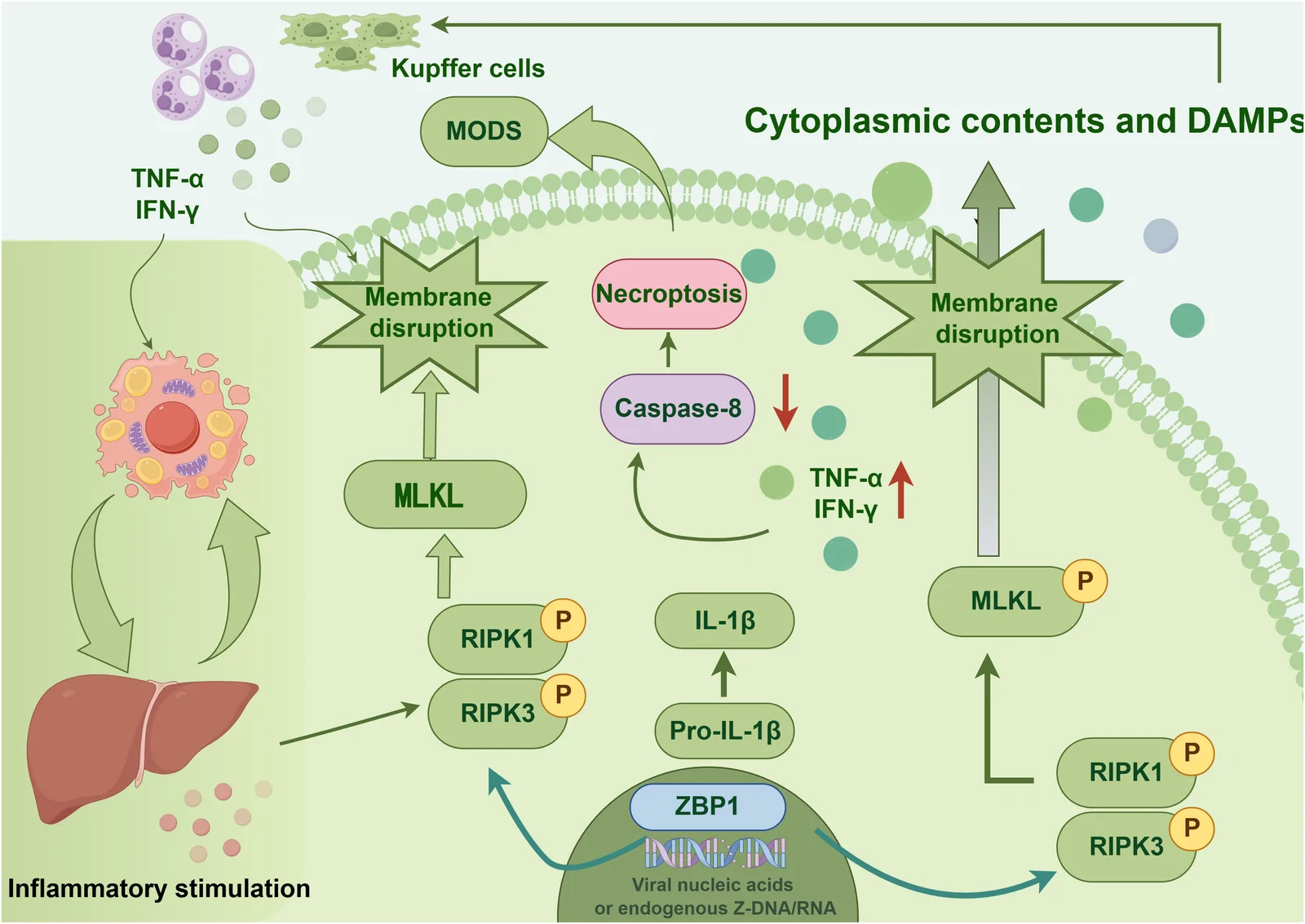
Potential inflammatory mechanisms linking cytokine signaling to hepatocyte necroptosis and necroinflammation. Schematic representation of how inflammatory cues precipitate hepatocyte necroptosis and amplify systemic injury. Initiation/priming by cytokines: pro-inflammatory cytokines (notably TNF-α and IFN-γ) activate death-receptor and inflammatory signaling, creating conditions permissive for necroptosis, particularly when caspase-8 activity is reduced or inhibited, thereby releasing the RIP kinase axis from apoptotic checkpoints. Execution of necroptosis: RIPK1 and RIPK3 undergo phosphorylation and assemble into the necrosome, culminating in MLKL phosphorylation, oligomerization, and translocation to the plasma membrane. Activated MLKL disrupts membrane integrity, resulting in membrane rupture and leakage of intracellular contents. Necroinflammatory amplification: membrane disruption leads to the release of damage-associated molecular patterns (DAMPs) and cytoplasmic alarmins, which stimulate hepatic innate immune cells (e.g., Kupffer cells) and reinforce inflammatory cytokine production. In parallel, DAMP-driven inflammasome signaling promotes caspase-1 activation and processing of pro–IL-1β into mature IL-1β, further amplifying the inflammatory milieu. Upstream regulation: ZBP1 is depicted as a nucleic-acid sensor that can engage RIPK3-dependent necroptosis and coordinate inflammatory outputs, linking intracellular danger sensing to necroptotic and inflammasome pathways. Collectively, this feed-forward necroptosis–DAMP–cytokine loop propagates local liver injury and contributes to multiple organ dysfunction syndrome (MODS). TNF-α, tumor necrosis factor-α; IFN-γ, interferon-γ; DAMPs, damage-associated molecular patterns; RIPK1/3, receptor-interacting serine/threonine-protein kinase 1/3; MLKL, mixed lineage kinase domain-like pseudokinase; ZBP1, Z-DNA binding protein 1; IL-1β, interleukin-1β; MODS, multiple organ dysfunction syndrome.

The inflammatory characteristics of necroptosis suggest that it may participate in amplifying hepatic immune responses. Release of DAMPs following membrane rupture can activate Kupffer cells and infiltrating immune cells, potentially enhancing cytokine production and sustaining local inflammation Similar mechanisms have been described in other sterile inflammatory conditions (67). However, whether this inflammatory amplification occurs in HS-ALI requires direct experimental confirmation.

Therefore, necroptosis should currently be considered a candidate inflammatory death pathway in HS-ALI rather than an established therapeutic target. Future studies using HS-specific models with temporal analysis of RIPK3/MLKL activation and cell-type-specific approaches are necessary to clarify its biological significance. Current evidence does not establish whether necroptosis preferentially occurs at a later stage of HS-ALI; therefore, its temporal relationship with pyroptosis and ferroptosis remains unresolved.

### 4.4 Autophagy: a context-dependent stress-response pathway in HS-ALI

Autophagy is a highly conserved intracellular degradation pathway responsible for removing damaged proteins and organelles and maintaining cellular metabolic homeostasis (**Figure 6**). Through autophagosome formation and lysosomal degradation, autophagy contributes to protein quality control, mitochondrial turnover, and adaptation to cellular stress (69). During heat stress, increased oxidative burden and mitochondrial injury may activate autophagic responses as an adaptive mechanism (70). In liver cells, autophagy can promote clearance of damaged mitochondria through mitophagy, reduce oxidative stress accumulation, and preserve energy metabolism. Experimental studies in non-HS liver injury and cellular stress models have demonstrated that intact autophagic activity may exert protective effects by maintaining hepatocyte survival and limiting inflammatory activation (71). However, these observations provide mechanistic context rather than direct evidence for a protective role of autophagy in HS-ALI.

**Figure 6:**
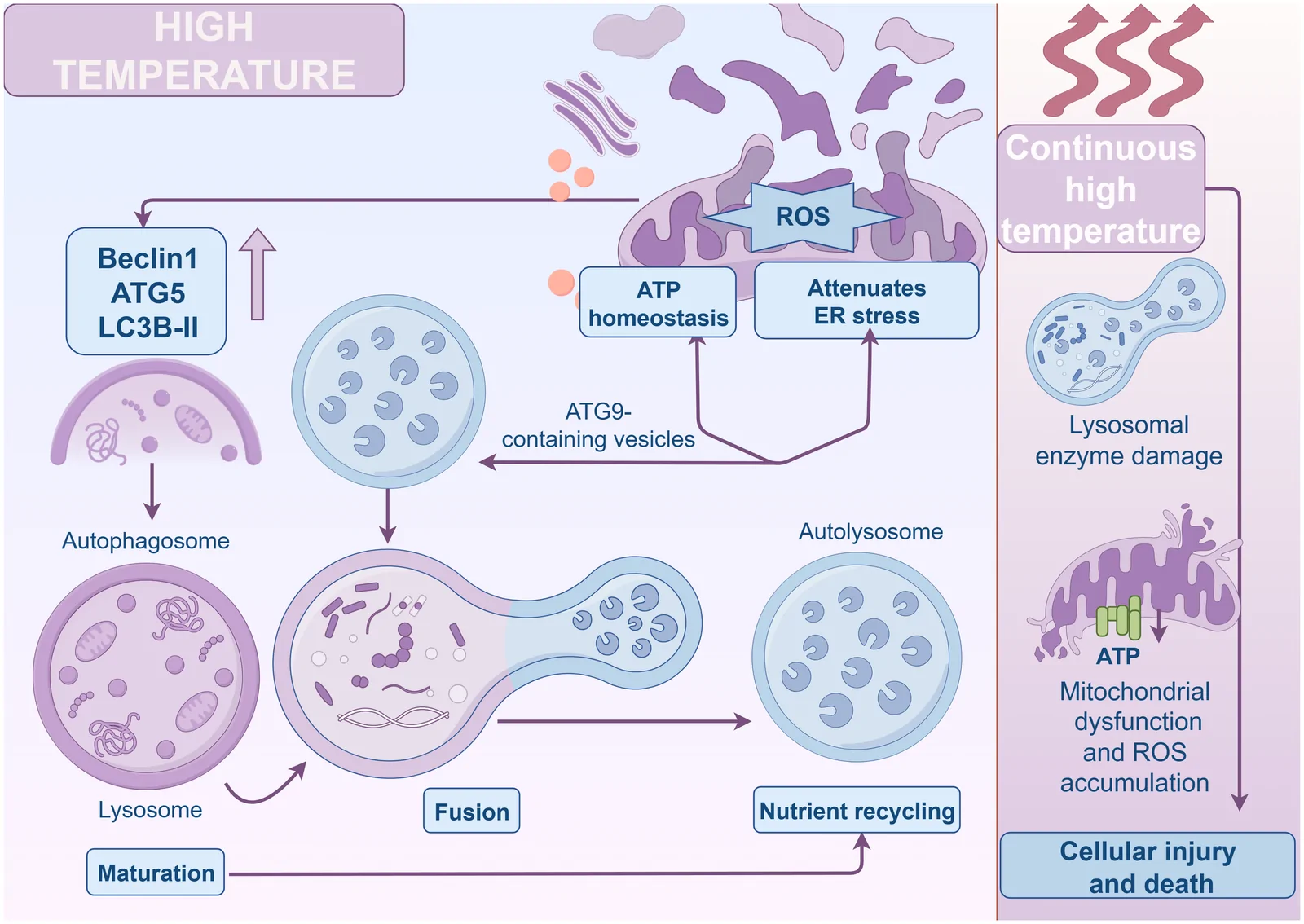
Context-dependent regulation of autophagic flux during heat stress: adaptive responses and potential maladaptation. Schematic depiction of how heat stress reshapes autophagy and determines cell fate. Adaptive autophagy under transient/controlled hyperthermia (left): heat exposure upregulates core autophagy machinery (BECN1/Beclin 1, ATG5, and LC3B-II), promotes autophagosome formation and maturation, and supports autophagosome–lysosome fusion to generate autolysosomes. Delivery of membrane components via ATG9-containing vesicles facilitates autophagosome biogenesis, enabling nutrient recycling that helps maintain cellular bioenergetics (stable ATP) and alleviates proteostasis burden, thereby dampening endoplasmic reticulum (ER) stress/UPR signaling. Potential maladaptation under sustained heat stress (right): prolonged thermal stress may impair lysosomal integrity, disrupt autophagic flux, promote energy depletion, and increase ROS accumulation. These alterations may reduce the cytoprotective capacity of autophagy and contribute to cellular injury. Importantly, this schematic illustrates context-dependent changes in autophagic function rather than a validated stage-specific transition from protective autophagy to autophagic cell death in HS-ALI. BECN1, Beclin 1; ATG, autophagy-related gene; LC3B-II, lipidated LC3; ER, endoplasmic reticulum; UPR, unfolded protein response; ROS, reactive oxygen species.

Despite these findings, the role of autophagy in HS-ALI remains insufficiently characterized. Although heat stress is known to influence autophagy-related signaling pathways, direct evidence defining whether autophagy is protective or detrimental in HS-induced liver injury is currently lacking (70). In other pathological contexts, excessive activation of autophagy may reflect severe cellular stress rather than a direct cause of cell death (71). Importantly, autophagy interacts extensively with other regulated cell death pathways. For example, ferritinophagy, a selective autophagic degradation process involving ferritin turnover, can increase intracellular iron availability and potentially promote ferroptosis under specific conditions (66). However, whether ferritinophagy contributes meaningfully to ferroptosis or liver injury during HS has not been directly established. Conversely, autophagy may also limit oxidative injury by removing damaged mitochondria and reducing ROS accumulation. These opposing effects highlight the context-dependent nature of autophagy regulation (70, 71). Current evidence does not support the assumption that autophagy inevitably progresses into “autophagic cell death” during HS-ALI. Instead, autophagy should be viewed as a dynamic stress-response pathway whose biological outcome depends on the intensity and duration of heat exposure, cellular energy status, and interaction with other injury pathways (71). Although autophagy may exert context-dependent protective or detrimental effects, current evidence does not establish that it follows a predictable stage-specific transition during HS-ALI.

### 4.5 A hypothetical interaction network of programmed cell death pathways in HS-ALI

Heat stroke-associated acute liver injury (HS-ALI) is characterized by the coexistence of thermal stress, oxidative imbalance, inflammatory activation, mitochondrial dysfunction, and metabolic disturbance. Under these complex stress conditions, multiple programmed cell death (PCD) pathways may be simultaneously activated and interact with each other. Rather than functioning as independent linear pathways, pyroptosis, ferroptosis, necroptosis, and autophagy-related processes may constitute an interconnected regulatory network that influences hepatocyte survival, inflammatory amplification, and tissue repair (35, 58–60) (**Figure 7**). Importantly, the currently proposed “programmed cell death storm” model remains a conceptual hypothesis rather than an experimentally validated temporal sequence. Although individual PCD pathways have been implicated in different forms of acute liver injury, direct evidence defining their chronological activation pattern, relative contribution, and cell-specific functions in HS-ALI is still lacking. Therefore, the previously proposed sequence of “early pyroptosis, intermediate ferroptosis, and late necroptosis” should not be regarded as an established temporal sequence and remains to be tested in dedicated longitudinal HS-specific models.

**Figure 7:**
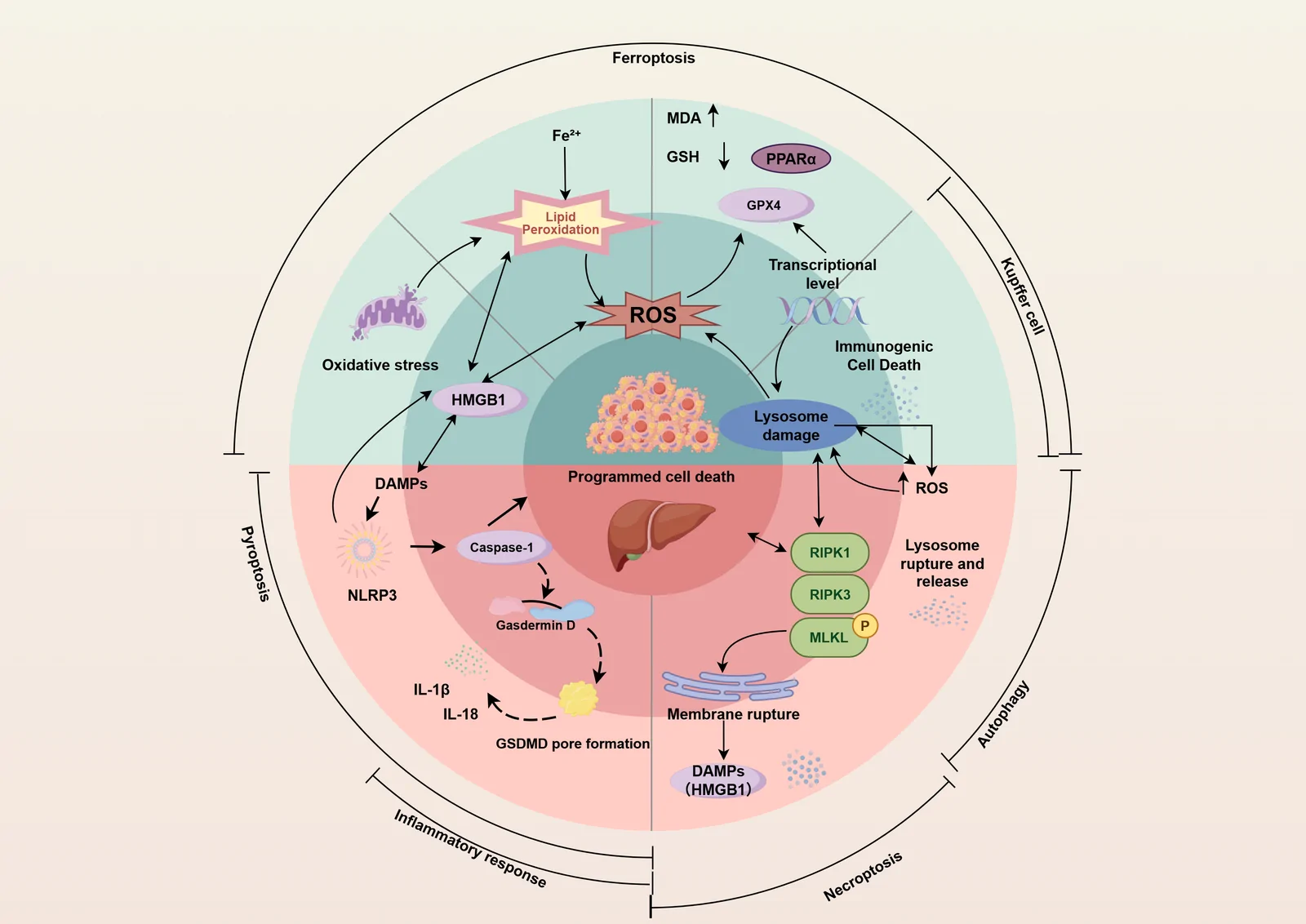
Crosstalk among regulated cell death pathways in the liver. Schematic model illustrating how ferroptosis, pyroptosis, necroptosis, and autophagy/lysosomal stress intersect in the hepatic microenvironment and converge on hepatocyte injury. These programs share key hubs—ROS/redox imbalance, lipid peroxidation, lysosomal dysfunction, and inflammatory signaling—forming feed-forward loops that amplify tissue damage. Ferroptosis is driven by labile Fe²^+^, GSH depletion, and GPX4 loss, leading to lipid peroxidation (e.g., MDA increase) and bioenergetic failure. Pyroptosis is initiated by LPS/DAMPs and inflammasome activation (e.g., NLRP3), promoting IL-1β/IL-18 maturation and inflammatory cell lysis. Necroptosis engages RIPK1-dependent signaling and results in membrane disruption with release of alarmins such as HMGB1, further aggravating inflammation. Autophagy modulates redox and lysosomal homeostasis but may become maladaptive under sustained stress, thereby potentiating other death programs. The figure illustrates potential mechanistic crosstalk and does not imply a validated temporal sequence among these pathways during HS-ALI. RCD/PCD, regulated/programmed cell death; ROS, reactive oxygen species; DAMPs, damage-associated molecular patterns; LPS, lipopolysaccharide; GSH, glutathione; GPX4, glutathione peroxidase 4; MDA, malondialdehyde; NLRP3, NOD-like receptor family pyrin domain-containing 3; IL, interleukin; HMGB1, high mobility group box 1; RIPK1, receptor-interacting serine/threonine-protein kinase 1; PPARα, peroxisome proliferator-activated receptor alpha.

Rather than reiterating the individual execution mechanisms described above, the major significance of this framework lies in the potential convergence and cross-regulation of different PCD pathways. Shared upstream stresses, including mitochondrial dysfunction, oxidative imbalance, inflammatory signaling, and metabolic disturbance, may alter cellular susceptibility to multiple death programs, while processes such as ferritinophagy may provide direct molecular links between autophagy and ferroptosis (66). Importantly, these interactions are likely to be cell- and context-dependent, and their temporal organization in hepatocytes, Kupffer cells, endothelial cells, and infiltrating immune cells during HS-ALI has not yet been established. The clinical implications of this PCD interaction framework remain largely exploratory. Importantly, HS-specific experimental studies now provide direct mechanistic support for both pyroptotic and ferroptotic signaling in HS-associated liver injury, including activation of the NLRP3–caspase-1–GSDMD pathway and experimental evidence of ferroptosis-dependent hepatic injury (58, 59, 64). However, mechanistic relevance should not be equated with biomarker validity Neither circulating GSDMD-N nor GPX4 has been adequately validated for diagnosis, prognosis, or therapeutic stratification in human HS-ALI. These molecules should therefore be regarded as experimental pathway indicators until their clinical performance is established in well-characterized HS cohorts. Future studies integrating single-cell transcriptomics, spatial transcriptomics, proteomics, and longitudinal sampling in HS models may help determine whether specific PCD pathways dominate at different stages of HS-ALI progression. Such investigations will be essential for transforming the current conceptual PCD network into clinically meaningful strategies for risk stratification and targeted intervention. Accordingly, the current PCD interaction framework should be interpreted as a hypothesis-generating model that organizes existing mechanistic observations rather than as a validated chronological cascade of cell death events in HS-ALI.

## 5 The hepatic immune cell network and inter-organ communication in HS-ALI

Heat stroke-associated acute liver injury (HS-ALI) is increasingly recognized as a systemic inflammatory disorder rather than a direct consequence of hepatocyte damage alone (3, 35).Following severe heat exposure, the liver acts as both a target organ and an immunological regulator, integrating signals derived from thermal stress, metabolic disturbance, intestinal barrier dysfunction, and systemic inflammatory activation (3, 35, 72). Hepatic immune responses involve coordinated interactions among Kupffer cells, liver sinusoidal endothelial cells (LSECs), infiltrating neutrophils, monocytes/macrophages, and lymphocyte populations (59, 73, 74) (**Figure 8**). However, the current understanding of immune regulation in HS-ALI remains incomplete Much of the mechanistic evidence derives from sepsis, ischemia–reperfusion injury, and other inflammatory liver disease models (73, 75).Although these studies provide biologically plausible mechanisms, their direct relevance to human HS-ALI requires further validation. Therefore, immune pathways discussed below should be interpreted as potential contributors rather than fully established HS-specific mechanisms (35).

**Figure 8:**
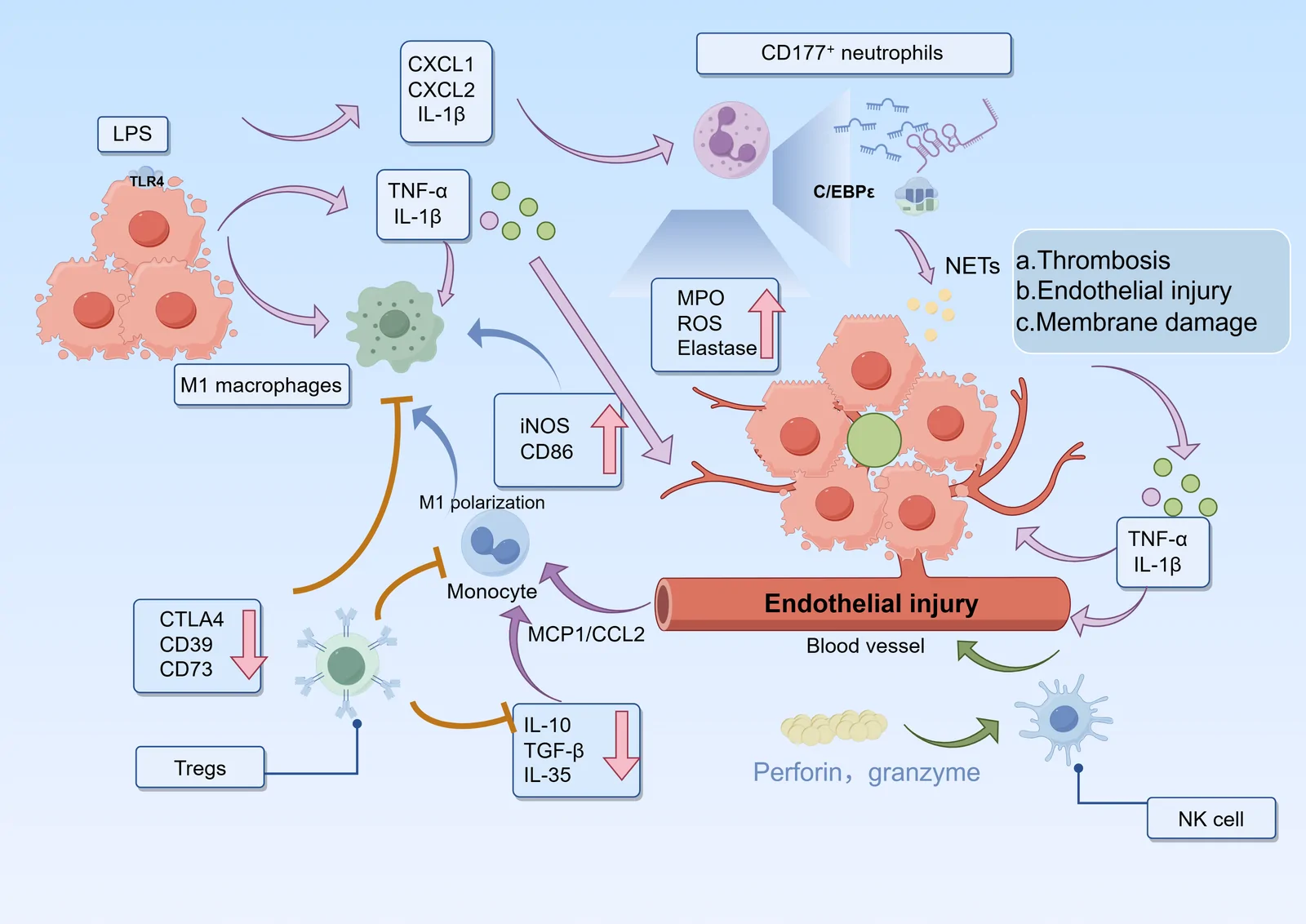
Proposed immune crosstalk contributing to hepatic endothelial injury in heat stroke. Schematic summary of the multicellular immune network that amplifies hepatic microvascular injury during heat stress. Hyperthermia-associated danger signals activate Kupffer cells through TLR4, inducing chemokines/cytokines (e.g., CXCL1/2, IL-1β) that recruit and activate neutrophils. Neutrophils promote endothelial damage via MPO-derived oxidants, ROS, elastase, and NET formation (C/EBPϵ-associated neutrophil program), contributing to thrombogenesis, endothelial disruption, and membrane injury. In parallel, M1-polarized macrophages (iNOS^+^/CD86^+^) reinforce inflammation through TNF-α and IL-1β, while endothelial MCP-1/CCL2 supports monocyte recruitment and feed-forward inflammatory signaling. Tregs (CTLA4–CD39–CD73 axis) counter-regulate these responses through IL-10, TGF-β, and IL-35, whereas NK cells aggravate vascular dysfunction via perforin/granzyme-mediated cytotoxicity. The imbalance between pro-inflammatory and regulatory circuits culminates in hepatic endothelial injury and microcirculatory failure. NETs, neutrophil extracellular traps; TLR4, toll-like receptor 4; DAMPs, damage-associated molecular patterns; MPO, myeloperoxidase; MCP-1/CCL2, monocyte chemoattractant protein-1.

### 5.1 Neutrophil recruitment and potential mechanisms of tissue injury

Neutrophils are among the earliest innate immune populations recruited during systemic inflammatory responses following heat stroke (HS) (74, 76). In experimental models, severe hyperthermia induces endothelial activation, increased vascular permeability, and release of inflammatory mediators, which may promote neutrophil recruitment into injured organs, including the liver (36, 76).Chemokines belonging to the CXC family, particularly CXCL1 and CXCL2, together with their receptor CXCR2, are well-established regulators of neutrophil trafficking in inflammatory disorders (36). However, most mechanistic evidence regarding the CXCL–CXCR2 axis originates from models of bacterial infection, sterile inflammation, or ischemia–reperfusion injury, and its specific contribution to human HS-associated acute liver injury (HS-ALI) remains incompletely defined.

Evidence from experimental heat stroke models suggests that neutrophil accumulation occurs in multiple organs during the early phase of thermal injury and is associated with enhanced production of inflammatory cytokines and oxidative stress (74, 76). In particular, IL-1β-driven inflammatory signaling has been reported to promote CXCL1 expression and neutrophil recruitment in heat stroke-related tissue injury models, suggesting that neutrophils may participate in the amplification of early inflammatory responses. Nevertheless, whether neutrophil infiltration represents a primary determinant of hepatic injury or a secondary consequence of systemic inflammation requires further investigation.

Neutrophils may contribute to liver injury through several potential mechanisms. First, excessive activation of neutrophils can induce the formation of neutrophil extracellular traps (NETs), which are composed of extracellular DNA decorated with myeloperoxidase (MPO), neutrophil elastase (NE), and other granular proteins (73, 77). NETs are increasingly recognized as important mediators of immunothrombosis and endothelial injury in sepsis and other inflammatory diseases (77–79). In the context of HS, severe hyperthermia, endothelial dysfunction, and oxidative stress provide a biologically plausible environment for NET formation (74, 80); however, direct clinical evidence demonstrating NET-dependent hepatic injury in HS-ALI remains limited. Second, activated neutrophils release ROS, MPO, and proteolytic enzymes (81),which may aggravate hepatocyte oxidative damage, mitochondrial dysfunction, and microvascular injury (73, 78). These mechanisms have been extensively demonstrated in sterile inflammatory liver injury and ischemia–reperfusion models (82), but their relative contribution to HS-induced hepatic injury requires validation in dedicated HS models. Neutrophils also interact with resident hepatic immune cells, particularly Kupffer cells and hepatic sinusoidal endothelial cells. NET components and neutrophil-derived inflammatory mediators may further stimulate macrophage activation, thereby enhancing the production of IL-1β, TNF-α, and other inflammatory mediators (75, 78). Such reciprocal communication between neutrophils and macrophages has been demonstrated in systemic inflammatory conditions; however, whether this immune amplification loop represents a dominant pathway in HS-ALI remains unclear.

Recent studies have highlighted the potential heterogeneity of neutrophil populations, including CD177-expressing subsets with enhanced antimicrobial and inflammatory functions (74, 83). Although CD177^+^ neutrophils and NET formation have been implicated in inflammatory diseases such as sepsis and acute respiratory distress syndrome, their specific involvement in HS-ALI has not yet been sufficiently characterized (74, 83, 84). Future studies integrating single-cell transcriptomics and spatial profiling may help determine whether distinct neutrophil subsets contribute to different stages of HS-induced liver injury. From a therapeutic perspective, targeting neutrophil-mediated inflammation is an attractive but currently unvalidated strategy CXCR2 inhibitors, NET formation inhibitors, and modulators of neutrophil activation have shown protective effects in experimental inflammatory models (74, 80); however, their efficacy, safety, and optimal timing in HS-ALI remain unknown Considering that neutrophils also contribute to host defense and tissue repair (73), indiscriminate suppression of neutrophil activity may carry potential risks. Therefore, future interventions should focus on selectively regulating pathogenic neutrophil activation rather than complete immune inhibition.

### 5.2 Recruitment and functional reprogramming of monocytes/macrophages in HS-ALI

Monocytes and macrophages are key regulators of hepatic inflammation during acute injury (75, 85). In response to tissue stress, circulating monocytes can be recruited into the liver through chemokine-mediated mechanisms, particularly the CCL2–CCR2 axis (86), and subsequently acquire inflammatory or reparative phenotypes depending on the local microenvironment. In various models of acute liver injury, recruited CCR2^+^ monocytes contribute to inflammatory amplification by producing cytokines, reactive oxygen species (ROS), and nitric oxide (NO), while resident Kupffer cells function as early sensors of danger signals and regulators of hepatic immune responses. These mechanisms have been demonstrated in conditions such as ischemia–reperfusion injury and toxin-induced liver injury (75, 85). In HS-ALI, severe hyperthermia, oxidative stress, and possible intestinal-derived microbial signals may activate hepatic macrophage populations (59, 87). However, direct evidence defining monocyte recruitment dynamics, macrophage subsets, and functional polarization patterns in HS remains limited (59, 88, 89). Therefore, whether HS induces a classical “M1-dominant” macrophage response remains uncertain.

Rather than a simple M1/M2 dichotomy, macrophage responses in acute liver injury are increasingly recognized as a spectrum of activation states influenced by cytokines, metabolic conditions, and tissue repair signals (89–91). During excessive inflammatory activation, macrophages may enhance tissue damage through production of TNF-α, IL-1β, IL-6, ROS, and inflammatory mediators (75, 85). Conversely, reparative macrophage programs may contribute to resolution of inflammation and tissue recovery (89, 90, 92). Therefore, macrophages in HS-ALI should be considered dynamic regulators whose functions may vary according to disease stage and cellular context. Future studies using single-cell transcriptomics and spatial profiling are required to determine whether specific macrophage subsets are associated with progression or recovery from HS-associated liver injury (59, 88, 93).

### 5.3 Contribution of T cells, NK cells, and adaptive immune responses

Although innate immune activation represents the earliest response during HS, adaptive immune cells may also influence disease progression through regulation of inflammation and tissue repair (88, 93, 94). Heat stroke induces systemic immune alterations, including changes in circulating lymphocyte populations, suggesting that adaptive immunity participates in the host response to extreme thermal stress (95, 96) (97). Experimental evidence indicates that lymphocyte deficiency can influence susceptibility to heat stress. In immunodeficient mouse models, absence of functional T cells was associated with altered inflammatory responses and increased mortality following heat exposure, suggesting that T cells may contribute to immune regulation during HS (94). However, whether these effects are mediated through direct hepatic immune responses or systemic immune dysregulation remains unclear.

Regulatory T cells (Tregs) play important roles in maintaining immune tolerance and controlling excessive inflammation. In several inflammatory liver diseases, impaired Treg activity has been associated with uncontrolled immune activation. However, the specific role of Tregs in HS-ALI has not been fully established. Current evidence does not support a definitive conclusion regarding whether Treg dysfunction promotes hepatic injury or represents a compensatory response to systemic inflammation. Natural killer (NK) cells are another immune population involved in tissue surveillance and inflammatory regulation NK cells can release cytotoxic molecules, including perforin and granzymes, and produce cytokines such as IFN-γ that influence macrophage activation (98). Clinical observations in heat stroke patients have demonstrated alterations in lymphocyte subsets and immune activation profiles (95, 96), suggesting involvement of cytotoxic lymphocyte responses (88, 93). Nevertheless, direct evidence linking NK-cell-mediated cytotoxicity to hepatocyte injury in HS-ALI remains insufficient. Overall, adaptive immune cells likely participate in shaping the inflammatory landscape of HS (88, 93); however, their precise contribution to liver-specific injury remains poorly defined. Future investigations integrating immune phenotyping, single-cell sequencing, and longitudinal clinical sampling are needed to clarify whether T cells and NK cells represent pathogenic drivers, regulatory populations, or secondary responders during HS-ALI.

## 6 Clinical diagnosis, classification, biomarkers and translational research

Heat stroke-associated acute liver injury (HS-ALI) is a rapidly progressive form of hepatic dysfunction characterized by substantial clinical heterogeneity (3).Unlike many chronic liver diseases, HS-ALI develops within a short time frame and frequently occurs together with systemic inflammatory response, coagulation abnormalities, kidney injury, and neurological dysfunction (3, 7).Therefore, early identification of high-risk patients and accurate evaluation of disease progression remain major clinical challenges (30, 99–101). Currently, diagnosis of HS-ALI relies primarily on clinical history, biochemical evidence of hepatocellular injury, and assessment of hepatic synthetic function. However, no universally accepted HS-specific diagnostic criteria or validated risk stratification system is currently available (30). Most clinical decisions continue to depend on dynamic monitoring of conventional liver function parameters and evaluation of multi-organ dysfunction (7, 99, 102).

### 6.1 Clinical manifestations and conventional assessment

The typical laboratory pattern of HS-ALI is characterized by rapidly increasing serum aminotransferases, reflecting extensive hepatocellular injury (7, 99). In severe cases, alanine aminotransferase (ALT) and aspartate aminotransferase (AST) concentrations may increase to several thousand or even tens of thousands of IU/L (7).AST elevation may occur earlier than ALT elevation, reflecting mitochondrial injury and systemic tissue damage; however, these patterns are not specific to HS and may also occur in ischemic hepatitis and other forms of acute liver injury (99). Progressive impairment of hepatic synthetic function represents an important indicator of poor prognosis (103). Prolonged prothrombin time (PT), increased international normalized ratio (INR), hypofibrinogenemia, hyperbilirubinemia, and development of hepatic encephalopathy indicate progression toward acute liver failure (ALF). Previous clinical studies have demonstrated that severe HS may rapidly progress to ALF and is associated with high mortality, occasionally requiring emergency liver transplantation. Dynamic assessment of platelet count, fibrin degradation products, D-dimer, fibrinogen, and coagulation time may provide additional information regarding the evolution of coagulation disturbances and disseminated intravascular coagulation (DIC) (52, 104, 105).

Inflammatory markers, including C-reactive protein (CRP), procalcitonin (PCT), and cytokines such as IL-6, may reflect systemic inflammatory activation; however, their diagnostic specificity for HS-ALI remains limited because similar alterations occur in sepsis, trauma, and other critical illnesses (99). Therefore, these indicators should currently be considered supportive markers rather than HS-specific diagnostic tools (30). Clinical classification of HS-ALI remains another unresolved issue. Although patients can present with different temporal patterns, including early severe injury and delayed progression, standardized staging systems comparable to those used in other forms of acute liver failure have not yet been established (3). Future multicenter studies are required to define clinically meaningful HS-ALI phenotypes.

### 6.2 Emerging biomarkers and multi-omics-based approaches

Advances in transcriptomics, proteomics, and metabolomics have created new opportunities to identify biomarkers associated with HS-ALI (106–108). However, most candidate biomarkers remain at the experimental stage and require validation in well-characterized clinical cohorts (30, 109). Mechanistic indicators reflecting specific regulated cell death pathways have attracted considerable attention (110). GSDMD-N reflects proteolytic activation of GSDMD during pyroptosis, whereas GPX4 is a central component of the antioxidant defense against ferroptotic lipid peroxidation. Importantly, HS-specific experimental studies support the involvement of both pyroptotic and ferroptotic pathways in HS-associated liver injury (59, 60, 64, 111). However, pathway involvement does not establish these molecules as clinical biomarkers. Whether circulating GSDMD-N or GPX4 measurements provide independent diagnostic or prognostic information in human HS-ALI remains unknown. Therefore, they should currently be interpreted as experimental mechanistic indicators rather than validated clinical biomarkers (30). Metabolomic studies have identified metabolic disturbances associated with heat stress, including alterations in lipid metabolism, mitochondrial energy metabolism, and purine metabolism (106). These findings are consistent with the central role of oxidative stress and mitochondrial dysfunction in HS pathophysiology. However, metabolic signatures identified in animal models require further validation before clinical application (30).

Circulating microRNAs represent another promising biomarker class because of their stability and tissue-associated expression patterns miR-122 and miR-192 have been extensively studied as markers of hepatocellular injury in various acute liver diseases (112). Nevertheless, their specificity for HS-ALI remains unclear because similar alterations occur in drug-induced liver injury, viral hepatitis, and ischemic hepatic injury. Therefore, future studies should determine whether combinations of circulating nucleic acids, inflammatory mediators, and metabolic signatures can improve early diagnosis and prognostic prediction Integrating multi-dimensional biomarkers with clinical parameters may represent a future direction for precision medicine in HS-ALI (106) (113). Machine-learning approaches combining physiological variables, laboratory indicators, and omics-derived signatures may facilitate early identification of patients at risk for ALF or MODS (106). However, large prospective cohorts and external validation are required before these approaches can be implemented in clinical practice.

### 6.3 Prospects for artificial intelligence and precision medicine in HS-ALI

Rapid advances in artificial intelligence (AI), multi-omics technologies, and computational medicine have created new opportunities to improve the diagnosis and prognostic evaluation of critically ill patients. However, compared with other acute liver diseases, the application of intelligent technologies in heat stroke-associated acute liver injury (HS-ALI) remains at an early exploratory stage, mainly because of the limited availability of large-scale clinical cohorts, heterogeneous disease phenotypes, and the absence of standardized biomarker systems (30). Currently, clinical evaluation of HS-ALI primarily relies on dynamic monitoring of physiological parameters, conventional biochemical indicators, and organ dysfunction scores (99). Integrating these multidimensional data through machine-learning algorithms may improve early risk stratification by identifying complex patterns that are difficult to recognize using traditional statistical approaches (110, 111, 114). Previous studies in intensive care populations have demonstrated that AI-based models incorporating continuous physiological monitoring and laboratory variables can assist in predicting deterioration and mortality in critically ill patients (115); however, their specific performance in HS-ALI requires prospective validation (116). Multi-omics integration represents another promising strategy for deciphering the biological heterogeneity of HS-ALI (106, 117). Transcriptomic, proteomic, and metabolomic analyses may help identify molecular signatures associated with oxidative stress, mitochondrial dysfunction, immune activation, and programmed cell death (59, 106). Nevertheless, most current omics findings originate from experimental models or small observational studies, and whether these molecular signatures can be translated into clinically applicable diagnostic tools remains uncertain (30, 106).Large multicenter cohorts integrating clinical phenotypes with molecular profiles will be necessary to establish clinically meaningful HS-ALI subtypes (118).

Single-cell RNA sequencing (scRNA-seq) has substantially advanced our understanding of cellular heterogeneity in liver diseases by identifying distinct immune and parenchymal cell populations involved in injury and repair processes (119). Studies in acute liver injury and inflammatory liver diseases have demonstrated that single-cell approaches can reveal disease-associated immune cell states and intercellular communication networks (119, 120). However, direct application of scRNA-seq to human HS-ALI remains limited, and its current role should be considered hypothesis-generating rather than clinically established. Similarly, computational imaging approaches, including radiomics and AI-assisted image analysis, have shown potential in detecting subtle structural alterations in various liver diseases. Nevertheless, imaging-based assessment of HS-ALI is challenging because the disease progresses rapidly and imaging abnormalities may lag behind biochemical and molecular changes. Therefore, future applications should focus on integrating imaging information with physiological monitoring and molecular biomarkers rather than relying on imaging alone. Ultimately, the future of precision medicine in HS-ALI will likely depend on establishing an integrated “clinical–molecular–computational” framework. Such a system may combine continuous physiological monitoring, conventional laboratory parameters, immune-metabolic biomarkers, and multi-omics signatures to enable early identification of high-risk patients and individualized therapeutic strategies. However, before clinical implementation, these approaches require rigorous validation through multicenter prospective studies, standardized data platforms, and clinically interpretable prediction models (111).

## 7 Treatment strategies and intervention targets

Treatment of heat stroke-associated acute liver injury (HS-ALI) should be interpreted according to the level of clinical evidence supporting each intervention (2, 35).At present, rapid active cooling and supportive critical care remain the foundations of heat stroke management, whereas no pharmacological therapy has been established specifically for HS-ALI (2, 121). Current evidence-based guidelines recommend immediate active cooling, with cold- or ice-water immersion providing the most rapid reduction in core temperature when feasible Pharmacological approaches aimed at reducing body temperature are not supported by current evidence (121).

Once HS-ALI progresses toward acute liver failure (HS-ALF), management largely follows established principles for acute liver failure, including hemodynamic stabilization, neurological and metabolic monitoring, correction of organ dysfunction, and timely assessment for extracorporeal support or liver transplantation when appropriate (7, 24, 122, 123). Importantly, these clinically established supportive measures should be distinguished from mechanism-targeted interventions that remain investigational in HS-ALI (2, 35) (**Figure 9**). Emerging strategies targeting oxidative stress, inflammatory signaling, ferroptosis, pyroptosis, necroptosis, and the gut–liver axis have shown biological or therapeutic effects predominantly in experimental models (58, 60, 68, 124).Accordingly, these approaches should currently be considered preclinical or hypothesis-generating strategies rather than established treatments for patients with HS-ALI (2, 35). The relative evidence levels of representative biomarkers, supportive interventions, and mechanism-targeted strategies are summarized in **Table 1**.

**Figure 9:**
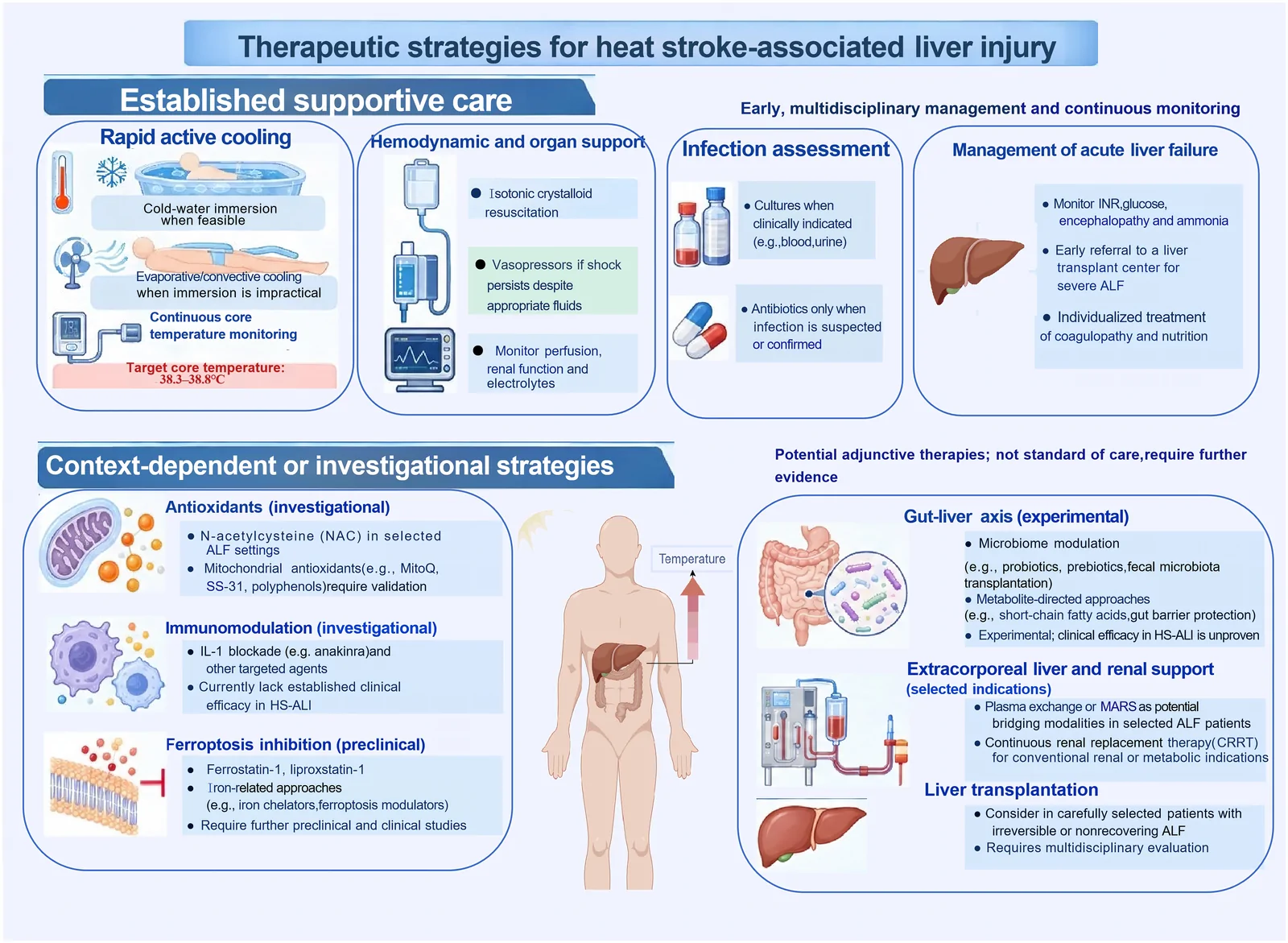
Multimodal management and candidate intervention targets for heat stroke and heat stroke–associated liver injury. Schematic overview of a multimodal clinical management framework integrating rapid temperature control, supportive care, and mechanism-informed adjunctive interventions. Early cooling is prioritized to limit ongoing thermal injury, followed by hemodynamic stabilization with fluid resuscitation and vasopressors as needed to maintain adequate perfusion. For heat stroke–associated hepatic dysfunction, measures include close metabolic monitoring (e.g., glucose), early nutritional support, and correction of coagulopathy when indicated. Given the frequent diagnostic overlap between sterile systemic inflammation and infection, infection workup (e.g., cultures) and antimicrobial therapy are applied based on clinical context. Potential adjunctive strategies targeting downstream injury mechanisms are highlighted, including antioxidants to mitigate oxidative stress, experimental modulators of inflammatory mediators, and inhibitors of regulated cell death pathways (e.g., ferroptosis) under investigation. Interventions aimed at preserving the gut–liver axis and escalation to extracorporeal liver support (e.g., MARS, plasma exchange) or renal replacement therapies (e.g., CRRT) are depicted as bridge therapies in selected severe cases, with liver transplantation reserved for irreversible failure. MAP, mean arterial pressure; ALF, acute liver failure; DIC, disseminated intravascular coagulation; ROS, reactive oxygen species; MARS, molecular adsorbent recirculating system; PE, plasma exchange; CRRT, continuous renal replacement therapy.

### 7.1 Rapid cooling and hemodynamic support: the cornerstone of HS management

Rapid active cooling is the cornerstone of heat stroke management and should be initiated immediately once HS is suspected (121, 125). The 2025 Society of Critical Care Medicine guideline recommends active cooling over passive cooling and identifies cold- or ice-water immersion as the modality achieving the most rapid reduction in core temperature when feasible. Importantly, current evidence does not support pharmacological interventions intended to reduce body temperature in patients with heat stroke (121).Cold-water immersion is particularly applicable to exertional heat stroke (EHS), in which rapid heat removal is critical and logistically feasible in many athletic and military settings (126) (121). For classic heat stroke (CHS), which predominantly affects elderly individuals or patients with impaired thermoregulatory capacity, cooling strategies may require greater individualization because underlying cardiovascular disease and reduced physiological reserve influence tolerance to rapid temperature reduction. Therefore, differences between EHS and CHS should be considered when selecting cooling approaches (2, 125). Current recommendations emphasize immediate cooling without delaying treatment for laboratory confirmation (121, 125).A commonly proposed therapeutic goal is to reduce core temperature rapidly to below 39 °C while avoiding excessive cooling-related complications (125).

Hemodynamic stabilization is another essential component of HS-ALI management (2, 125). Heat exposure induces peripheral vasodilation, dehydration, endothelial dysfunction, and impaired microcirculatory perfusion, potentially worsening hepatic ischemia and inflammatory injury (2, 3). Early administration of isotonic crystalloid solutions is recommended for volume restoration. Patients with persistent hypotension despite adequate fluid resuscitation may require vasopressor support, with norepinephrine commonly used to maintain adequate systemic perfusion (127). However, optimal hemodynamic targets in HS remain insufficiently defined Excessive vasoconstriction may theoretically aggravate microvascular dysfunction; therefore, individualized assessment of organ perfusion rather than blood pressure normalization alone should guide treatment decisions.

### 7.2 Supportive management and organ protection in HS-associated acute liver failure

When HS-ALI progresses to acute liver failure (HS-ALF), management largely follows established principles for acute liver failure (7, 122, 123). The primary objectives include prevention of cerebral edema, maintenance of metabolic homeostasis, correction of coagulation abnormalities, and management of extrahepatic organ dysfunction. Hypoglycemia is a frequent complication of severe liver dysfunction because impaired hepatic gluconeogenesis reduces glucose availability. Therefore, continuous monitoring of blood glucose and timely glucose supplementation are essential Hepatic encephalopathy and cerebral edema represent major causes of mortality in ALF Management includes neurological monitoring, avoidance of factors that increase intracranial pressure, optimization of ventilation and circulation, and administration of osmotic agents such as mannitol or hypertonic saline when clinically indicated (123, 128).

Nutritional management should aim to maintain adequate energy and protein supply. In contrast to earlier recommendations for protein restriction, current ALF recommendations favor sufficient protein administration with individualized adjustment according to metabolic status because excessive restriction may aggravate malnutrition and impair recovery (128).Because HS-ALF frequently occurs together with renal failure, coagulation disorders, and systemic inflammatory responses, multidisciplinary management involving intensive care specialists, hepatologists, nephrologists, and transplant teams is essential (7, 24, 122).

### 7.3 Infection surveillance and antimicrobial management

Heat stroke (HS) is primarily a sterile inflammatory disorder induced by extreme thermal stress rather than an infectious disease (125, 129). However, severe HS, particularly cases complicated by acute liver failure (HS-ALF) and multiple organ dysfunction syndrome (MODS), is frequently accompanied by immune dysregulation, intestinal barrier impairment, and increased susceptibility to secondary infections (88, 129).Therefore, infection surveillance is an important component of supportive management, although antimicrobial therapy should not be routinely initiated in the absence of clinical or microbiological evidence of infection (121).

Experimental and clinical studies suggest that hyperthermia-associated intestinal epithelial injury may increase intestinal permeability and facilitate translocation of bacterial products, including lipopolysaccharide (LPS), into the portal circulation (124, 130, 131). However, the contribution of endotoxin translocation to HS-associated liver injury remains mechanistically plausible but has not been fully validated in human HS cohorts (9, 35). Importantly, activation of inflammatory pathways induced by LPS, such as TLR4-dependent signaling, has been extensively characterized in sepsis and other inflammatory liver injury models, but whether these mechanisms represent dominant drivers in HS-ALI requires further investigation (9, 47). Clinically, secondary infections have been reported in patients with severe HS, particularly those requiring prolonged intensive care support (129, 132). Early reports described positive blood cultures in fatal HS cases, suggesting that bacterial infection may occur during the later course of disease rather than representing the initial trigger. Therefore, persistent fever despite adequate cooling, unexplained hemodynamic deterioration, increasing inflammatory markers, or new organ dysfunction should prompt evaluation for infection, including microbiological cultures and appropriate imaging when indicated (133). When bacterial infection or septic complications are confirmed, antimicrobial therapy should follow standard principles for critically ill patients, with adjustment according to microbiological findings and organ function. Nevertheless, unnecessary empirical broad-spectrum antibiotics should be avoided because antibiotic exposure may further disturb gut microbiota and potentially aggravate intestinal barrier dysfunction. Future studies are required to determine whether biomarkers distinguishing sterile HS inflammation from secondary infection can improve antimicrobial decision-making.

### 7.4 Antioxidant therapy and mitochondrial protection strategies

Oxidative stress and mitochondrial dysfunction are important components of HS-induced cellular injury (3, 35).During severe heat exposure, excessive reactive oxygen species (ROS) production, impaired mitochondrial respiration, and energy depletion may contribute to hepatocyte dysfunction (3, 58). However, most mechanistic evidence regarding antioxidant interventions originates from experimental models of ischemia–reperfusion injury, drug-induced liver injury, or other oxidative stress-associated diseases, and their therapeutic efficacy in HS-ALI remains unconfirmed (35).

N-acetylcysteine (NAC) occupies an intermediate position between established acute liver failure therapy and an HS-specific investigational intervention. In a randomized controlled trial, intravenous NAC improved transplant-free survival in patients with early-stage non-acetaminophen acute liver failure, supporting its clinical use in selected ALF settings (134). However, evidence specific to heat stroke-associated ALI/ALF remains limited. In the Acute Liver Failure Study Group series of eight patients with heat stroke-associated ALI/ALF, three patients received NAC, but the observational design and small sample size precluded assessment of treatment efficacy (7). Accordingly, NAC should not currently be regarded as an established HS-specific therapy, although its broader role in acute liver failure provides a rationale for further evaluation in this setting (7, 134).

Several mitochondria-targeted antioxidants, including MitoQ and SS-31, have demonstrated protective effects against mitochondrial ROS accumulation and membrane dysfunction in experimental models of oxidative injury. Nevertheless, these compounds have not been evaluated in HS-ALI models with sufficient evidence to support clinical application. Their potential role should therefore be considered hypothesis-generating rather than established. Similarly, natural compounds such as resveratrol and melatonin have been reported to regulate antioxidant pathways including SIRT1/Nrf2 signaling and suppress inflammatory activation in various experimental liver injury models. However, their effects in HS-ALI remain largely unexplored. Given the complex pathophysiology of HS involving thermal injury, inflammation, coagulation abnormalities, and metabolic failure, antioxidant therapy alone is unlikely to reverse disease progression without simultaneous correction of temperature elevation, circulatory instability, and organ dysfunction (2, 35). Future research should focus on identifying HS-specific oxidative stress signatures and determining whether antioxidant intervention can modify clinically meaningful outcomes, rather than solely improving biochemical markers in experimental models.

### 7.5 Targeting inflammatory responses and immune dysregulation

Given the inflammatory mechanisms discussed above, cytokine- and inflammasome-directed interventions have been proposed as potential therapeutic strategies; however, HS-ALI-specific therapeutic evidence remains limited and is largely experimental (35, 59, 60).The IL-1 signaling pathway has attracted considerable attention because IL-1β is a potent mediator of systemic inflammation and endothelial activation (60). The IL-1 receptor antagonist anakinra has demonstrated anti-inflammatory effects in several hyperinflammatory syndromes, including macrophage activation syndrome and cytokine storm-related diseases (135–137). However, clinical evidence supporting its use in heat stroke remains lacking, and controlled studies are required before translation into HS management. Similarly, TNF-α and IL-6 represent potential therapeutic targets based on their established roles in systemic inflammation (9, 99). Anti-TNF therapy and IL-6 receptor blockade have shown efficacy in autoimmune and cytokine-driven diseases, but immunosuppression during heat stroke may theoretically increase susceptibility to secondary infections. Consequently, cytokine-directed therapies should currently be considered experimental approaches rather than standard treatments for HS-ALI (35).

Glucocorticoids have historically been used in severe inflammatory conditions; however, their role in heat stroke remains controversial. Current evidence does not support routine corticosteroid administration for heat stroke because immunosuppression may increase infectious complications and has not demonstrated clear survival benefits (121). Management should therefore focus primarily on rapid cooling, organ support, and correction of physiological disturbances rather than nonspecific immune suppression (2, 121).

### 7.6 Targeting ferroptosis and other programmed cell death pathways

Based on the PCD mechanisms discussed in Section 3, pharmacological modulation of ferroptosis and other regulated cell death pathways has emerged as a potential therapeutic strategy (58, 60, 68). However, most intervention studies have been conducted in experimental liver injury models, and direct evidence supporting PCD-targeted therapy in HS-ALI remains limited (58, 59). Accordingly, these approaches should currently be considered preclinical rather than established therapeutic strategies (35). Similarly, iron chelation therapy may theoretically reduce iron-mediated oxidative injury, particularly in patients with concomitant rhabdomyolysis and systemic iron overload. Nevertheless, clinical application requires careful evaluation because iron metabolism abnormalities during critical illness are complex and iron restriction may interfere with essential physiological processes. Other programmed cell death pathways, including pyroptosis and necroptosis, have also been proposed as therapeutic targets (60, 64, 68). Inflammasome inhibitors, caspase pathway modulators, and RIPK1/RIPK3/MLKL pathway inhibitors have shown protective effects in experimental inflammatory injury models (60, 68). However, similar to ferroptosis, their relevance to HS-ALI requires validation in heat stroke-specific animal models and prospective clinical studies (9, 35, 58, 68, 124). Therefore, programmed cell death-targeted therapies should currently be regarded as hypothesis-generating strategies. Future studies should determine whether specific molecular signatures can identify patients who may benefit from pathway-specific intervention.

### 7.7 Gut–liver axis protection and microbiota-based interventions

The gut–liver axis has emerged as a potential contributor to HS-associated systemic inflammation (9, 35, 124). Hyperthermia can impair intestinal epithelial integrity, increase intestinal permeability, and facilitate the entry of microbial products into circulation (124, 130, 131). Nevertheless, the extent to which intestinal bacterial translocation directly drives HS-ALI in humans remains uncertain (9, 35, 124). Evidence from critical illness and sepsis suggests that intestinal microbiota disruption and increased endotoxin exposure can amplify systemic inflammation through immune activation pathways. However, these findings should not be directly extrapolated to HS without further validation (129). Strategies targeting intestinal barrier preservation, including nutritional support, microbiota modulation, and prevention of excessive antibiotic exposure, may theoretically be beneficial. Probiotics and microbiota-directed interventions have demonstrated immunomodulatory effects in critically ill populations, although their efficacy in heat stroke remains unproven (138–140).

Short-chain fatty acids (SCFAs), microbially derived metabolites with important roles in immune regulation and epithelial integrity, represent another potential therapeutic avenue. Experimental studies indicate that SCFAs can influence macrophage polarization, regulatory T-cell responses (141), and intestinal barrier function (138–140, 142, 143).Whether these mechanisms can be therapeutically exploited in HS-ALI requires further investigation. At present, clinical management should emphasize early enteral nutrition, avoidance of unnecessary antibiotics, and maintenance of intestinal integrity rather than routine microbiome-targeted therapy.

### 7.8 Extracorporeal liver support in HS-associated acute liver failure

When heat stroke-associated acute liver injury progresses to acute liver failure (HS-ALF), extracorporeal support may be considered as a bridging strategy while hepatic recovery is awaited or liver transplantation is being evaluated (24, 144–146). The rationale extends beyond simple removal of hepatotoxic metabolites. Severe HS-ALF is characterized by systemic inflammation, coagulopathy, metabolic derangement, renal dysfunction, and accumulation of circulating toxins; therefore, extracorporeal therapies may theoretically be beneficial by removing soluble inflammatory mediators and toxic metabolites, correcting metabolic disturbances, and, in the case of plasma exchange, replacing depleted coagulation factors (145, 147). Nevertheless, evidence specifically supporting these interventions in HS-ALF remains limited, and most clinical data are extrapolated from acute liver failure of other etiologies (144, 148).

Plasma exchange (PE), particularly high-volume plasma exchange (HVP), has the strongest clinical evidence base among extracorporeal support approaches in acute liver failure (147, 149, 150). In addition to removing circulating bilirubin, ammonia-associated metabolites, damage-associated molecules, and inflammatory mediators, PE simultaneously replaces plasma proteins, albumin, and coagulation factors, providing a mechanistic rationale for its use in patients with severe coagulopathy and systemic inflammatory activation (145, 147). In a multicenter randomized controlled trial involving 182 patients with acute liver failure, HVP significantly improved transplant-free survival and was associated with reductions in systemic inflammatory response syndrome and Sequential Organ Failure Assessment scores, suggesting that modulation of innate immune activation may contribute to its clinical effects (147). HS-specific evidence for PE is far more limited A published case report described complete recovery of a 73-year-old patient with non-exertional heat stroke-associated acute liver failure after high-volume plasma exchange; however, isolated case experience cannot establish treatment efficacy or identify which patients are most likely to benefit (148).

Albumin-based dialysis systems, particularly the Molecular Adsorbent Recirculating System (MARS), are designed to remove albumin-bound substances such as bilirubin, bile acids, aromatic metabolites, and other circulating toxins that are inefficiently cleared by conventional dialysis (148). By reducing the circulating toxic burden, MARS may improve biochemical parameters, hepatic encephalopathy, and hemodynamic disturbances. However, evidence for a survival benefit remains uncertain (144, 151). In a randomized controlled trial of 102 patients with acute liver failure, MARS did not significantly improve 6-month survival compared with conventional therapy, although interpretation was limited by the high rate and early timing of liver transplantation (151). Expert consensus therefore considers albumin dialysis primarily as a supportive or bridging approach in carefully selected patients rather than a definitive therapy (144). Continuous renal replacement therapy (CRRT) should be distinguished from liver-specific extracorporeal support because its primary indications in severe HS are acute kidney injury, rhabdomyolysis-associated metabolic disturbances, electrolyte abnormalities, volume dysregulation, and severe acidosis (7, 122). In acute liver failure, CRRT may provide additional benefit by facilitating ammonia clearance and maintaining metabolic homeostasis, which may be particularly relevant in patients at risk of hepatic encephalopathy and cerebral edema (145, 152). In a large Acute Liver Failure Study Group cohort, CRRT was associated with a greater reduction in serum ammonia and lower 21-day transplant-free mortality compared with no renal replacement therapy, although the observational design precludes a definitive causal conclusion (152). Thus, in HS-ALF, CRRT should primarily be initiated according to renal and metabolic indications, with ammonia control representing a potential additional benefit rather than evidence of direct hepatoprotective efficacy. Overall, extracorporeal therapies in HS-ALF should currently be viewed as supportive or bridging interventions rather than HS-specific definitive treatments (144). PE has the strongest evidence base in general acute liver failure, whereas evidence for MARS remains mixed and CRRT is primarily indicated for renal, metabolic, and ammonia-related support (147, 151, 152). Dedicated prospective studies in HS-ALF are needed to define patient selection, optimal timing, treatment intensity, and effects on transplant-free survival.

### 7.9 Liver transplantation in HS-associated acute liver failure

Liver transplantation (LT) represents the definitive rescue therapy for a small subset of patients with HS-associated acute liver failure who fail to recover despite maximal supportive management (7, 24, 122, 153). However, determining the optimal timing of transplantation is particularly challenging in HS-ALF because severe biochemical and coagulation abnormalities may still be reversible, whereas unnecessary early transplantation exposes potentially recovering patients to major surgery and lifelong immunosuppression (24, 154, 155). Previous reports have documented spontaneous recovery despite fulfillment of conventional transplantation criteria, highlighting the limitations of applying prognostic models developed for other causes of acute liver failure directly to HS-ALF (154, 156, 157). The most informative clinical evidence regarding LT selection in this setting comes from the multicenter retrospective study by Ichai et al. (24). Among 24 patients with exertional heat stroke (EHS)-related severe acute liver injury and a prothrombin time (PT) below 50%, 15 improved with medical therapy alone and survived, whereas nine were initially listed for emergency LT. Importantly, five of the nine listed patients were subsequently removed from the waiting list because PT began to recover, predominantly between days 2 and 3 after heat stroke. Ultimately, only four patients underwent LT; transplantation was performed in the setting of persistent PT <10% approximately 3 days after EHS together with more than three organ dysfunctions Three of the four transplanted patients were alive at 1 year. Histological examination of the explanted livers demonstrated massive or submassive necrosis with limited regenerative activity (24).These observations suggest that the trajectory of hepatic synthetic function may be more informative than a single extreme laboratory value when assessing transplantation candidacy in HS-ALF (24, 158). In particular, recovery of PT during the first several days may identify patients with substantial regenerative potential, whereas persistently profound coagulopathy accompanied by progressive multiorgan dysfunction should prompt urgent reassessment for transplantation (24). Nevertheless, the PT <10% threshold and associated organ dysfunction profile reported by Ichai et al. should be regarded as clinically informative observations rather than validated universal transplantation criteria, because they were derived from a relatively small retrospective cohort (24). To facilitate comparison between mechanistic candidates and clinically applicable approaches, the current evidence levels of representative biomarkers and therapeutic or supportive interventions relevant to HS-ALI are summarized in **Table 1**.

## 8 Discussion and conclusion

Heat stroke-associated acute liver injury is a severe manifestation of systemic heat stress and remains a major determinant of morbidity and mortality in severe heat stroke. Current evidence indicates that HS-ALI arises from complex interactions among thermal damage, circulatory disturbance, oxidative stress, immune dysregulation, endothelial dysfunction, and metabolic imbalance. Recent studies have expanded understanding beyond the traditional concept of nonspecific thermal necrosis by identifying potential roles for inflammatory signaling, mitochondrial injury, intestinal barrier dysfunction, and programmed cell death pathways. However, many mechanistic findings are derived from experimental models or related inflammatory diseases, and their specific contribution to human HS-ALI requires further validation. Clinically, early cooling, hemodynamic stabilization, intensive organ support, and timely recognition of acute liver failure remain the foundation of treatment. Although emerging strategies targeting oxidative stress, inflammatory pathways, ferroptosis, and immune modulation provide promising directions, none have yet been established as HS-specific therapies.

Future progress will depend on integrating mechanistic research with clinical investigation through standardized disease models, multicenter cohorts, advanced biomarker validation, and precision medicine approaches. Particular attention should be given to differences between exertional and classic heat stroke, as well as vulnerable populations including elderly and pediatric patients. Ultimately, shifting HS-ALI management from empiric supportive care toward evidence-based, individualized intervention will require close collaboration among basic scientists, intensivists, hepatologists, and translational researchers.
